# The institutionalization of parasitology in São Paulo: the contributions of Émile Brumpt between 1913 and 1948

**DOI:** 10.1590/S0104-59702024000100042en

**Published:** 2024-10-11

**Authors:** Denis Guedes Jogas

**Affiliations:** i Postdoctoral fellow, Department of Research/Casa de Oswaldo Cruz/Fiocruz. Rio de Janeiro – RJ – Brazil denis.jogas@hotmail.com

**Keywords:** Émile Brumpt (1877-1951, São Paulo School of Medicine and Surgery, Parasitology, Leishmaniasis, Chagas disease

## Abstract

This article draws on a large volume of documents retrieved from the historical archives of Institut Pasteur, in Paris, the Manguinhos library of the Oswaldo Cruz Foundation, and the Hemeroteca Digital library run by National Library of Brazil to analyze the links between the institutionalization of parasitology in São Paulo and the presence of the French physician Émile Brumpt at the São Paulo School of Medicine and Surgery in its early years. Bringing to light information from previously unresearched or little-known primary sources, this article contributes to the historiography of exchanges between French and Brazilian scholars and the institutional memory of the Faculty of Medicine, University of São Paulo.

Brumpt, assistant [of] Blanchard wishes to teach Natural History at the new faculty, conditions? Also seek fitting professor [for] medical practice reply urgently, I will pay expenses. Arnaldo (Carvalho, 27 jan. 1913).

On April 2, 1913, one of the oldest and greatest desires harbored by a significant (and politically influential) part of the São Paolo middle classes began to take shape and be realized.^1^ With the passing of state law n.1357 on December 19, 1912, and its regulation by executive order n.2344, of December 31, 1913, the first activities of the new São Paulo School of Medicine and Surgery (Escola de Medicina e Cirurgia de São Paulo) could commence. The inaugural lessons, given in the lecture hall of the Polytechnic School of São Paulo (Escola Politécnica de São Paulo), were in medical physics and natural history, given by Edmundo Xavier (1861-1933) and Celestino Bourroul (1880-1958), respectively.^2^

Notwithstanding the limited and improvised nature of the facilities,^3^ Arnaldo Augusto Vieira de Carvalho (1867-1920),^4^ an influential physician from São Paulo who was appointed the school’s first director,^5^ was in charge of ensuring the smooth running and organization of the new course (Mota, 2005; Fonseca, 2002; Marinho, Mota, 2012). Based on the powers described in the aforementioned law and executive order, he set about building a robust faculty composed of experienced Brazilian and foreign physicians, as well as acquiring “material indispensable for the assembly of the laboratories,” which was often purchased from abroad.^6^

The first foreign professor to be hired was Alexandre Joseph Émile Brumpt (1877-1951), who would be responsible for natural history.^7^ A notable disciple of Rafael Blanchard, founder of the Institute of Colonial Medicine in Paris,^8^ Brumpt, aged 36, had already made his name as a physician in his home country. Alongside the experience he had amassed at the Paris Faculty of Medicine (Faculté de Médecine de Paris) as an assistant, head of practical work, and associate professor [*professeur agrégé*], he had also led scientific expeditions to the tropics and published in 1910 the first edition of his *Précis de Parasitologie*, which was subsequently reedited (in 1913, 1922, 1927, 1936, and 1949). As such, he was a very influential figure not only among the São Paulo medical community, but across the whole of Latin America.

Getting Brumpt on board was a great boon for the nascent institution, whose very existence was being rivaled at the same time by another recently founded faculty of medicine in the city, albeit of a private nature. The arrival of acclaimed professors from abroad could be interpreted as a strategy employed by Carvalho and his group to beat its competitor (Mota, 2005; Teixeira, 2007). As we will see throughout this text, Brumpt’s participation in the early years of the state medical course had such repercussions that he was later remembered in its institutional memory as the founder of this field of medicine in the city, even though he spent only a short while lecturing on the course and was involved in some significant disagreements with a group of students.

This article explores some specific aspects of this history, such as the prior trajectory of Émile Brumpt, the circumstances that led to his being hired, the main activities he undertook while in São Paulo, some details of the termination of his contract after the outbreak of the First World War, and the active engagement of the Council of the Faculty of Medicine in the official celebrations for his scientific jubilee in 1948 in Paris.

## Connections between São Paulo and Paris: exchanges concerning human trypanosomiases and other parasitic diseases

The epigraph of this article was taken from a telegram Arnaldo Vieira de Carvalho sent to Firmiano de Morais Pinto (1861-1938), the commissioner of São Paulo state to France and Switzerland, in which he explained that “Blanchard’s disciple” had accepted the invitation to be professor of natural history at the new São Paulo School of Medicine. Carvalho also asked the commissioner to find out urgently what the parasitologist’s conditions were. In his reply, dated February 5, Pinto detailed Brumpt’s reply: “annual pay of 30,000 francs, a contract with the duration of two academic years, and four return tickets” between Paris and São Paulo. The letter also stated that if the contract were to be signed, Brumpt would be available to take up his position in Brazil in June of that year. As with the previous correspondence, a prompt reply was requested (Pinto, 5 fev. 1913).

With degrees in natural sciences, with emphasis in zoology, botany, and geology (1896), and physical sciences, with emphasis in general chemistry (1897), Émile Brumpt had doctoral degrees in natural sciences (1901) and medicine (1906).^9^ He became known in French and foreign medical circles after he took part in two expeditions to Africa. For the sheer size and scope of the undertaking, the first of these was a veritable epic, lasting 27 months (from June 1901 to March 1903). As a physician and naturalist participating in the mission led by the Viscount du Bourg de Bozas (who died during the journey of malaria and exhaustion), he crossed Equatorial Africa, entering the continent from Djibouti, in the Red Sea, and crossing as far as Brazzaville (today the capital of the Democratic Republic of Congo), then returning home by the Atlantic Ocean. Throughout almost the whole journey, he found his way using just a compass and collected abundant quantities of scientific material so he could “organize the practical work in parasitology at the Faculty of Medicine and at the Institute of Colonial Medicine”^10^ (Brumpt, 1934, p.30).

The second expedition, which set off just four months after his return to Paris, went to the French Congo. It lasted four months (from July to November 1903) and aimed to verify the hypothesis mooted during the first expedition that “the tsetse flies frequently [encountered] along the riverbanks were the agent of sleeping sickness” (Brumpt, 1934, p.30). Upon his return to France, Brumpt was accompanied by three Congolese colonists, Bobangui, Makaya, and Salomon, who had African trypanosomiasis (sleeping sickness). Treated at Hôspital des Dames Française, they were studied by European physicians until, one by one, they perished.^11^ The news that there were humans with the disease in Paris attracted researchers from elsewhere. A group from the London School of Tropical Medicine, made up of several of Britain’s foremost specialists, including Patrick Manson, James Cantile, George Low, and Louis Sambon, visited the hospital (Osborne, 2014, p.207, 208; A doença..., 20 fev. 1904).

It is important to remind ourselves that the medical world in the early 1900s had witnessed some significant breakthroughs in understanding the causes and nature of infectious and contagious diseases, especially in response to the microbial revolution, the doctrine of the single, individualized theological agent for each disease manifestation, and above all the mosquito theory, which stated that different species of blood-feeding insects were capable not only of transmitting diseases but also of being intermediate hosts of these diseases, with the microorganisms that were pathogenic to humans and animals playing an important role in their life cycle. These changes in the semiotics of diseases dynamized the relationships between different areas of natural history, such as entomology and zoology; they also lent biomedical research a new lease of life by providing a principle and/or a model for researches in different regions to respond to the challenges involved in piecing together the puzzle called “tropical diseases;” namely, relating clinical manifestations to their respective actors and pathogenic agents, especially in hot and humid climates (Benchimol, Sá, 2006; Benchimol, Jogas Jr., 2020; Jogas Jr., 2017, 2022; Caponi, 2002, 2003).

At around the same time that Brumpt was gaining a reputation for his research into sleeping disease, a student named Celestino Bourroul was defending his thesis, entitled “Mosquitoes of Brazil,” at the Faculty of Medicine of Bahia, northeastern Brazil. Studying under the supervision of Adolpho Lutz,^12^ he based his thesis on entomological research undertaken on the island of Itaparica. Thanks to the quality of his work, he was awarded a trip to Europe to continue his studies (Benchimol, Sá, 2006, p.58; Marinho, 2016, p.116). After a lengthy bureaucratic process, the payment of the funds was finally authorized by the federal Senate in the second half of 1908.^13^ Once in Europe, Bourroul spent time at different scientific institutions in France, Germany, and Austria. It is his time at the Institute of Colonial Medicine in Paris that is of particular interest for this article.^14^

Bourroul’s experience in Paris, before returning to São Paulo (in mid-1910), marked the beginning of a long relationship with Émile Brumpt. He became his main interlocutor during his time in Brazil and after his return to France, helping resolve certain bureaucratic loose ends related to the termination of his contract, as we will see later in this text.^15^ Furthermore, Celestino Bourroul was responsible for setting up and running the natural history course since the beginning of the academic year, on April 15, until Brumpt’s arrival in July of that year, and therefore one month after the date originally set for his arrival (Bourroul, 1913, p.1-13).

Carvalho saw the hiring of Brumpt as an important first step in the new course and therefore took great pains to ensure optimal conditions were available for the teaching activities, as well as granting him great freedom in organizing the course of study, as can be seen in early letters sent to the French parasitologist:

My dear and esteemed colleague,

I have just received a copy of the correspondence between yourself and the state commissioner in Paris and I am now sure of your collaboration in the organization of the new São Paulo School of Medicine. I have passed on your conditions to the department of the interior and believe there will be no problem in meeting them ...

I am most gratified to be able to count on you to ensure a good beginning for the teaching of medical sciences at our new school. Together with me, the entire medical community of São Paulo is overjoyed to be able to receive lessons from such a knowledgeable gentleman whose name has been so well known to them for so long ...

You will find a school that is taking its first steps and where the organization of the teaching of natural sciences (mainly parasitology) is reserved to yourself. I shall give you some explanations about what I understand this title to mean – natural sciences (mainly parasitology).

The study of natural sciences (botany, zoology) is done or should be done at the school. We are not responsible for anything except the parts of this discipline that are directly related to medicine, that is, to animal and plant parasitology.

Therefore, in the course, the professor will not be responsible for teaching general zoology or botany. He will simply give some lessons at the beginning to show the relationships between parasitology and botany and zoology and will then go into the specific study of parasites. You will be entirely responsible for deciding upon the distribution of the studies, the method, and the arrangement of the subjects, and I believe that for such an experienced a professor as yourself, there could be nothing more appropriate. Nonetheless, lessons should begin in April, but you will not have arrived by then. In the interim, I shall give a course of studies based on your treatise on parasitology [*Précis de Parasitologie*], which may be modified after your arrival.

Here in São Paulo, we do not have at our disposal everything needed for the teaching of your subject. It would therefore be helpful for the school if you would take it upon yourself to procure in France some indispensable objects. Would you be able to do this? If so, please be so kind as to calculate the sum required and notify me of it. I will obtain prompt authorization from the government … and will charge my correspondent … with facilitating the purchase and expedition of all of these things (Carvalho, s.d.).

Brumpt replied to the director listing the scientific material he deemed necessary, asked for some additional information about the faculty’s contact in Paris, and inquired whether it would be possible for him to return to France in October of the same year and whether any additional monies could be found for extra practical work in parasitology. In a letter dated April 14, 1913, Carvalho assured him that he should feel free to purchase whatever material he deemed necessary and to pick whomever he considered best equipped in Paris, explaining that “the time of your work goes from March to June and from July to November,” making it “impossible to return to France in October,” but stressing that he could go “in November after the exams.” He would therefore have “the months of December, January, February, and March to arrange his affairs and then return to his work here [in São Paulo].” As for the extra practical work, he said it was impossible to promise anything because he did not have a budget for this kind of work. “Nonetheless,” he continued, “bring your preparations and I promise to make every effort to ensure your comfort, as I wish most earnestly to see you amongst us because of our school and the many problems of our tropical diseases” (Carvalho, 14 abr. 1913).

Carvalho was good to his word. A letter from the school’s administration dated September 18, 1913, therefore after Brumpt’s arrival in Brazil, reads as follows:

According to your request and with the authorization of the secretary of the interior, I hereby notify that you are to receive 7,000 francs in 1913 and 10,000 francs in 1914 for practical work in the parasitology laboratory. This additional amount will be paid at the end of the academic year, that is, in November of the respective years (Carvalho, 18 set. 1913).

At this point in the narrative, it is worth asking one simple question: why did Émile Brumpt accept the invitation to work in São Paulo? It seems unlikely that it was because of the salary he was to receive from the state government or the friendship he had struck up with Celestino Bourroul; it is more likely that he was attracted for professional reasons related to two recent discoveries made by members of the Brazilian scientific community. The first was the description of *Trypanosoma cruzi* and American trypanosomiasis (Chagas disease) – the only other human trypanosomiasis to have been identified in the world aside from that associated with sleeping sickness (African trypanosomiasis), which Brumpt had made an object of study for many years.^16^ The second was the identification of protozoa of the genus *Leishmania* for the first time in the western hemisphere, in the town of Bauru in inland São Paulo state, and the controversy this sparked because it was a different species from the one that had been described previously and would therefore be responsible for a disease specific to South America: American cutaneous – or forest – leishmaniasis.^17^

Furthermore, in a speech he gave upon his scientific jubilee, in 1948, Brumpt acknowledged that his decision to take up the position in São Paulo had been influenced by Carlos José Botelho Jr., who at the time was studying medicine in Paris.^18^ A grandson of Antônio Carlos de Arruda Botelho, a baron, a viscount, and later the count of Pinhal, Botelho Jr. belonged to a family of some political weight in the state of São Paulo. By studying medicine in Paris, he was following in the footsteps of his father, Carlos José de Arruda Botelho, who had earned his degree there in 1878 before returning to São Paulo and practicing medicine at Santa Casa de Misericórdia hospital. He had also been active in several projects that had ultimately resulted in the creation of the São Paulo School of Medicine and Surgery.^19^ Additionally, a perfunctory look into the genealogy of Botelho Jr. in order to contextualize historically this person mentioned by Brumpt revealed that his aunt Cândida de Arruda Botelho – daughter of his grandfather’s second wife – was married to Firmiano de Moraes Pinto. We might therefore conjecture that these family connections may have been what enabled Botelho Jr. to study under the French parasitologist and exert a degree of influence – or some lobbying capacity – to convince him to take up the invitation to join the faculty at the new school of medicine.

## Émile Brumpt at the São Paulo School of Medicine and Surgery: research, teaching, and ultimately a split?

Émile Brumpt and his wife, Renée, disembarked from the Steamboat Frísia in the port of Santos on July 15, 1913. Waiting for him there were Celestino Bourroul and Léo Lopes de Oliveira,^20^ representing the faculty, as well as a “delegation of scholars ... who accompanied them to the capital city.” The train on which they were travelling reached Luz station, in downtown São Paulo, at 18h58. There, Brumpt was awaited by several state politicians and some distinguished figures from the medical community, including Arnaldo Viera de Carvalho, Edmundo Xavier, and Antônio Carini, alongside a large group of his future students, who gave him an “enthusiastic ovation” (Faculdade..., 16 jun. 1913).

Once the noise had died down, Synesio Rocha^21^ gave a welcome speech in French “on behalf of the young men of the São Paulo Faculty of Medicine and Surgery,” who were keen to “receive the great scientist admired by the whole of France; the most genuine representative of intellectual life in the country.” In turn, the Frenchman proffered a short speech of thanks and said he hoped to “correspond fully to the gracious invitation he had received from the São Paolo government.” He and his wife were then taken to Hotel Rôtisserie (Faculdade..., 16 jun. 1913; Hóspedes..., s.d.).

On July 19, 1913, or four days after his arrival, Émile Brumpt gave the inaugural lesson of his course in a session open to the public. The course would be given on Thursdays and Saturdays starting at 7h30 in the premises of the Alvares Penteado School of Trade (Escola de Comércio Alvares Penteado) (Notícias, 1913, p.1). As Celestino Bourroul had given the first classes, covering the topics until protozoa, he explained that he would begin with the study of arthropods. In the first lesson, however, he spoke more broadly about the connections established between “nature, environment, and parasitism … as it appears to us from the perspective of the animals and plants that are parasitic in man.” He suggested that with a few exceptions, such as hereditary syphilis, “men and animals generally come into the world free of infections,” receiving from nature and the environment “all the germs that affect them and ail them” (Brumpt, 21 jun. 1913, p.1). For the academic year, he planned to give a “more or less complete course in parasitology” because he deemed it “indispensable” for future physicians given the “intimate relationship between human medicine and veterinary medicine” (p.3).^22^

The practical and experimental nature of parasitology and its teaching was consistent with the values professed by Carvalho in the foundations of the new faculty, for which he “sought to confer a scientific and experimental basis for teaching, with emphasis on research and laboratory work” (Marinho, Mota, 2012, p.20). Another innovation was the more flexible set of admission criteria: not only were students of both sexes permitted, but 10% of all places were earmarked for poor students. Indeed, the legislation that created the school permitted several routes of admission to the foundation course, including people with “certificates granted by official high schools,” “graduates from any official higher education establishment in the State or the Republic,” and also students who wished to transfer from other Brazilian (Rio de Janeiro and Salvador) or even foreign faculties of medicine (Mota, 2005; Teixeira, 2007; Maia, 2017).

This range of acceptance criteria did not mean that the teaching was any less rigorous; in fact, it may have been a cause for the educational rigor, since the only precondition for accessing the general course was passing all the disciplines in the foundation course (Mota, 2005). Students even had to understand complex lessons in different foreign languages, such as parasitology, given in French, or anatomy, given in Italian by Alfonso Bovero, from the University of Turin (Souza Campos, 17 mar. 1948).

As analyzed by Mota (2005), even in the first year of the new course, the grading system and the high expectations of the foundation course sparked such strong protests amongst the students that the director ordered the closure of the school between August 16 and 21. According to a report published in *Correio Paulistano* newspaper on August 17, 1913, these students, “disgruntled with their low grades … decided to demonstrate their dissatisfaction” to professors Edmundo Xavier and Émile Brumpt by booing during their classes. They managed to put the plan into practice during a lesson given by the former and planned to repeat the protest in the lesson on parasitology. As “Dr. Brumpt was quick to react, as he is a guest, the students did not reproduce their demonstration of discontent.” Not satisfied with booing, the students decided to take some practical action and headed off to Carvalho’s private clinic with the aim of vandalizing it. When he heard of what was happening, Carvalho “took appropriate steps immediately” and was assured the support of the government to “take rigorous measures against the culprits,” setting up an inquiry to this end during the suspension of the course (Notas, 17 ago. 1913). However, he quickly realized that it would not be a simple task, because there were “doubts as to the people who were actually responsible for what had happened and also the surname of many of those students” (Mota, 2005, p.197).

In the end, the decision was taken not to punish any of the students and to reopen the doors of the course on August 23. Nonetheless,

the resumption of lessons was accompanied by more boos for professors Brumpt and Xavier, upon which the administration, already shaken, decided to close the Faculty [again] and hold a meeting of the Council, which, on September 3, according to article 210 of the regulation, suspended all the students who had attended the lesson that had been disrupted. In total, 95 students were punished, 59 of whom appealed to the Council. On September 16, the results of the midterm exams prompted new upset in Dr. Xavier’s class and a further 34 suspensions, as well as prohibiting the entry of the students. At that point, some of the students, considered by Dr. Arnaldo to be the “best in the faculty,” who had not taken part in the events, reported to the administration to repudiate the actions of their peers and express their support for the institution (Mota, 2005, p.196-197).

These upheavals resulted in a significant reduction in the size of the student body. Of the 180 students who had enrolled, just 70 sat the final exams; of these, 34 were admitted to the general course and 36 failed. This crisis also had major repercussions inside the Oswaldo Cruz Academic Center (Centro Acadêmico Oswaldo Cruz, Caoc). Founded in mid-1913, “it had students who were against the stance taken by the professors and the leadership, who were accordingly dismissed from the faculty and consequently from Caoc” (Mota, 2005, p.198). From this moment on, the leadership team took Caoc under its own sphere of influence and reorganized it under the chairmanship of Ernesto de Souza Campos,^23^ whereby it “took on the mission of collaborating with the administration and the plans set by the school” (Mota, 2005, p.199; Maia, 2017).

Aside from these mishaps with the students, Brumpt’s presence in the city was a cause of excitement amongst the São Paulo medical elite. At the session of the São Paulo Society of Medicine and Surgery (Sociedade de Medicina e Cirurgia de São Paulo) held on August 1, 1913, and the first after his arrival,^24^ he was named a corresponding member on the suggestion of the Italian physician Antonio Carini, who had served as the director of the Pasteur Institute of São Paulo (Instituto Pasteur de São Paulo) for some seven years and was one of the most active members of the society in this period (Teixeira, 1995, 2007; Ribeiro, 1996). At the following meeting, held on August 15, Brumpt was given the floor to give a speech on hygiene and parasitology, which lasted an hour and 10 minutes (Ata..., jun.-jul. 1913, p.4, 8).

Besides participating in this society’s meetings, Brumpt endeavored to forge partnerships with the local medical community that went beyond mere formality. One of the most significant activities he engaged in together with other physicians from the state was the medical/public health expeditions to inland parts of the state. These engagements, which, while expanding knowledge on the hottest research topics (such as leishmaniasis and Chagas disease), were part of a larger effort by the state’s political forces to take civilization to the hinterlands. By expanding coffee plantations, they aimed to establish and strengthen the presence of the state in the countryside, even if this entailed the genocide of indigenous people, such as was seen in the west of the state. At the time, these regions were framed by the political elites as a kind of territorial void (Mota, 2021; Bertolli Filho, 2021).^25^

One such contact was Alexandrino de Moraes Pedroso (1881-1922). With a degree from the University of Pennsylvania, he was in charge of the anatomical pathology laboratory at Santa Casa de Misericórdia and also taught histology and microbiology at the same establishment as Brumpt. Extant documents suggest that, alongside Celestino Bourroul, it was with Pedroso that Brumpt developed the most prolific professional ties during his first stay in Brazil.

In September 1913, Émile Brumpt and Alexandrino Pedroso kicked off (what should have been) a broad epidemiological study into the leishmaniases encountered in the region. Between September 9 and 30, they spent time in inland São Paulo state and the grasslands of Mato Grosso, marking what was to be just “the beginning of our investigations on Leishmaniasis americana” (Brumpt, Pedroso, 1913, p.100). They were interested in five aspects of the disease: (1) studies of typical cases (date of onset, location of ulcers on the body of the patient, patients’ opinions on the origin of the ulcers); (2) vectors (seasonal frequency, location, number of bites etc.); (3) reservoirs of the virus; (4) “innoculable” animals; and (5) experiments in how to solve the etiological problem (p.100, 101).

On the trip, they came across 65 cases of the disease, 90% of which they classified as mild, very similar to cutaneous leishmaniasis. In the remaining 10% of patients, the disease presented in malignant forms, with a particular tendency to propagate to the mucosal parts of the body, making it “a highly individualized ailment.” Its differentiated clinical and epidemiological characteristics prompted the researchers to call it “American forest leishmaniasis.” This name was designed to distinguish it from cutaneous leishmaniasis – endemic in highly populated areas – as it was found mostly in far-flung, sparsely inhabited areas near forests (Brumpt, Pedroso, 1913, p.97).

In their search for potential reservoirs, they examined a range of wild and domestic animals, including tapirs, deer, agoutis, dogs, horses, mules, donkeys, oxen, sheep, goats, cats, and pigs. They were unsuccessful, however, in capturing any “wild dogs – hard to hunt and little known, but certainly sensitive to the virus that produces the ulcers.” They hoped to have more success in a “future trip to disease-stricken zones.” Twice, they found ulcers on agoutis that they found suspicious, but they were unable to verify the existence of *Leishmania* in these wounds, which prevented them from confirming their hypothesis. Nonetheless, they did express the opinion that “if there are reservoirs, as we believe, they are constituted of wild animals – primarily the representatives of the genera *Canis* and *Aguti* [agouti]” (Brumpt, Pedroso, 1913, p.129, 130).

Another topic of interest to the researchers was identifying what transmitted American forest leishmaniasis. They collected and examined different species of worms, leeches, mites, ticks, lice, bedbugs, kissing bugs, mosquitoes, flies, blackflies (Simuliidae), horse and deer flies (tabanids), and sand flies (Phlebotominae) whenever they came across them. However, when they tried to make epidemiological correlations, they realized this was no easy task, because as the patients were “so used to forest life, they generally paid little attention to blood-sucking insects – to which, indeed, they had become accustomed,” making it “impossible, even for the most diligent observer, to know exactly what animal may bite and infect them in the place where the ulcers are reproduced” (Brumpt, Pedroso, 1913, p.102).

The animal they were looking for must be capable of inoculating the parasite in different parts of the human body, because there were so many cases of multiple ulcers on a single individual, and must also be active during the day, because they believed the disease was contracted during work in the forest. After giving the reasons why they ruled out each of the extensive list of species investigated, they reached the conclusion that horse flies (tabanids) could be responsible for transmitting the disease. They based this deduction on the fact that it was active at dawn and dusk, that it was a fast flyer, and that it was a tenacious biter, especially of human and animal heads and limbs (Brumpt, Pedroso, 1913, p.126, 127).

Some patients regarded sand flies, which as of the 1920s would be associated with the transmission of different clinical forms of leishmaniasis, as the “most feared animals in the forest” because they would bite repeatedly and painfully. However, they ruled out these blood-feeding flies as the vector of the forest disease because of their nocturnal habits and the recent conclusions published by Carlos Chagas after an expedition to the Amazon River valley^26^ (Brumpt, Pedroso, 1913, p.128).

On the very day he returned to São Paulo city, Émile Brumpt was invited by Ernesto de Souza Campos, president of Caoc, to give a talk at the headquarters of the Historical and Geographical Institute (Instituto Histórico e Geográfico). Brumpt accepted the invitation, and on October 25 he gave his lecture, entitled “Parasitological investigations in Bauru” (Centro..., 23 out. 1913; Conferência..., 26 out. 1913). At the event, Brumpt made his students the “invaluable offer” of a rare copy of his new work on parasitology (*Précis de pathologie*, 2nd edition), which had “not yet seen the light of publication” (Souza Campos, 29 set. 1913). It is possible that this invitation was an olive branch from the center’s new leadership team after the mishaps detailed above.

As arranged with Carvalho, Brumpt returned to Europe in November, where he stayed until March of the following year. On March 13, 1914, he was back in São Paulo set to begin his second academic year. Before the lessons began, he traveled with Bourroul to Salto Grande, near the border with Paraná state, because of the “many cases of malaria” that were appearing “abnormally” in the region’s coffee plantations. Brumpt took advantage of the trip to “collect materials for his parasitology course” (Impaludismo, 4 abr. 1914).

He went back to the same area of the state in May of that year, where he did new parasitology research and suggested some prophylactic measures to address the three main public health problems there, which “prevent progress, stall development, and can even stop what was sown in the farms from being harvested.” Specifically, these were: ancylostomiases, for which he advised “treating all sick individuals with thymol,” “building a latrine in each house,” and “expressly prohibiting defecation on the ground;” malaria, treating patients “assiduously ... with quinine” and fighting the disease in a “war against the transmitting mosquitoes;” and finally, on a lesser scale, forest ulcers (“Bauru ulcers, Northeast ulcers, angry wounds”), which, despite not knowing its vectors, seemed to “be caused by the large pools of standing water or water sources in the forest,” making it “wise to stay clear of them” (As fazendas..., 18 maio 1914).

**Figure 1:**
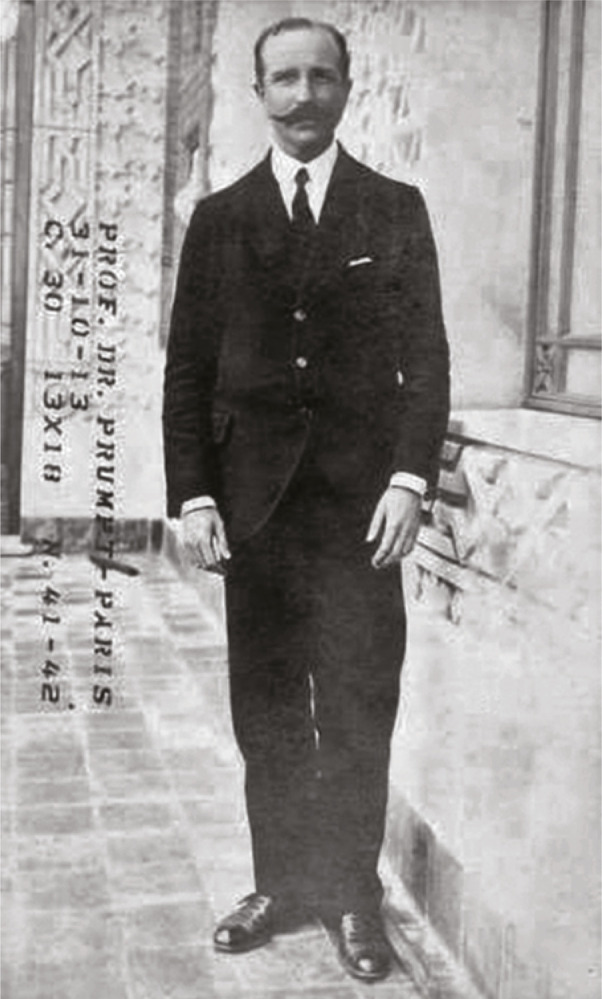
: Émile Brumpt at the Moorish pavilion of Instituto Oswaldo Cruz on October 30, 1913 (DAD/COC-Fiocruz, BR RJCOC 02-10-20-10-017-001)

In the same month, Brumpt embarked on another expedition with Alexandrino Pedroso. This time, they went to Albuquerque Lins, where, “in the big forest” in an area known to harbor an endemic, they studied “the suspected vector agents of American forest leishmaniasis” (Brumpt, 1934, p.32). The absence of any published results and the fact that the vectors of this group of diseases were not known until at least the 1920s indicate strongly that this second study trip was not as successful as the one conducted the previous year. The most significant happening in the second year of Brumpt’s time in São Paulo was his visit to Lassance (Minas Gerais state) “to see [Carlos] Chagas and there to study the disease that bears his name” (p.33). It would appear that planning for this trip had begun in late 1913, when Arnaldo de Carvalho had sent the following telegram to Oswaldo Cruz about his visit to Rio de Janeiro (Figure 1): “Professor Brumpt, chief of the mission organized by the São Paulo Faculty of Medicine to study the Bauru ulcer and Chagas disease, wishes to collect some work effects from Manguinhos, whither he is departing. Please facilitate this task for the distinguished scientist” (Notas, 30 out. 1913). This request was answered the following day by Oswaldo Cruz: “I shall be most satisfied [to] welcome Professor Brumpt at Manguinhos, providing whatever elements are within our reach” (Notas, 1 nov. 1913).

**Figure 2:**
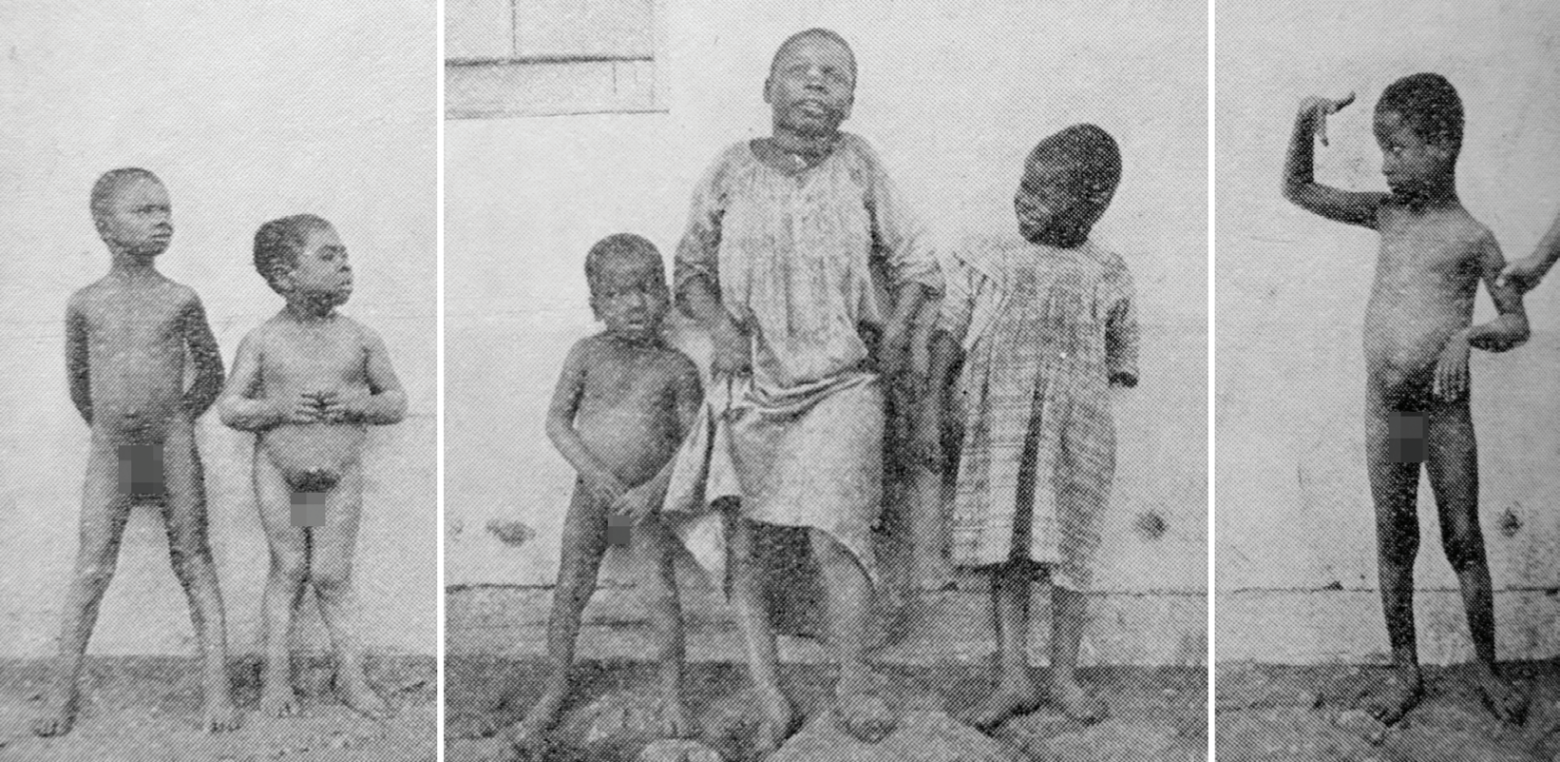
: Children with chronic Chagas disease photographed by Brumpt on his trip to Lassance (Brumpt, 1936, p.356)

**Figure 3:**
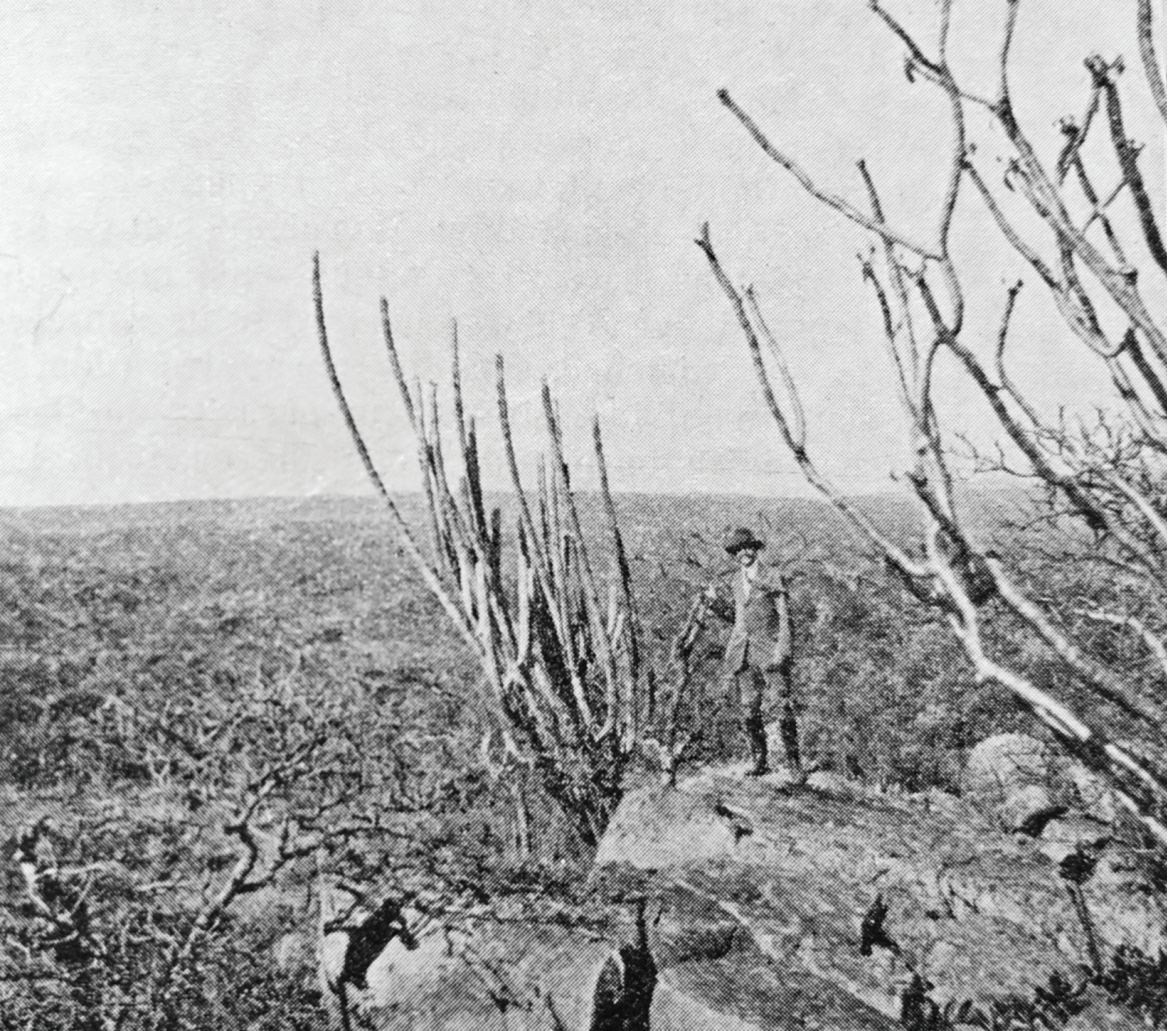
: Brumpt on Mount Cabral (800m altitude; 12km from Lassance), where *Triatoma chagasi* was encountered in 1913 (Brumpt, 1936, p.349)

In June 1914, Brumpt, Antonio Carini, and João Florencio Gomes (1886-1919) visited the village where Chagas had made his triple discovery of the vector, parasite, and clinical presentation of American trypanosomiasis. There, he could observe the great many patients who were treated at the hospital built by the Oswaldo Cruz Institute (Instituto Oswaldo Cruz) (Kropf, 2009), take photographs, which would illustrate future versions of his treatise on parasitology (Figure 2), and, accompanied by Gomes (Figure 3), undertake fruitful field trips into the regions adjacent to Lassance, which resulted in the description of a new species of kissing bug, *Triatoma chagasi*. Indeed, this finding led him to deduce that there may be a wild lifecycle of Chagas disease amongst wild mammals and to describe man as a secondary reservoir of this trypanosome, since the kissing bug was encountered in rock cavy burrows (*Kerodon rupestris*) in an uninhabited region that was naturally infected with *Tripanosoma cruzi* (Brumpt, Gomes, 1914, p.73-77; Brumpt, 1934, p.89).

In fact, Brumpt’s interest in American trypanosomiasis (and his collaboration with Brazilian researchers for its study) preceded his first visit to Brazil. In January 1912, he had partnered with the physician Pirajá da Silva, from the state of Bahia, at the Paris Faculty of Medicine to conduct detailed studies and experiments into the reproductive cycle and lifecycle of *Conorhinus megistus* (now *Panstrongylus megistus*), a vector of Chagas disease that has had captured in a village called Mata de São João in the state of Bahia. They also demonstrated the existence of a considerable quantity of trypanosomes in its waste and its capacity to infect laboratory animals. These observations served as a strong indication – which was subsequently confirmed – that the disease was not generally inoculated through a bite, but by contamination via its feces, with infection by biting occurring merely by accident (Brumpt, da Silva, 1912a, 1912b; Brumpt, 1934).

Throughout the first decades of the twentieth century, Émile Brumpt worked diligently on the study of Chagas disease, making significant contributions to knowledge about different facets of this trypanosomiasis (its clinical presentation, parasitological characterization, mode of transmission, description of new species, and the geographical distribution of its vectors and intermediate hosts) (Opinel, Gachelin, 2005). However, I would hazard to suggest that his greatest and longest lasting contribution to this pathology was his proposal of a new diagnostic method, which he devised in his latter months at São Paulo, and which continued to be relied upon until the emergence of molecular biology: xenodiagnosis. In broad terms, this method consists of ascertaining whether laboratory-bred kissing bugs (which were therefore free of infection) would be infected by biting an individual suspected of having the disease (Brumpt, 1914).

In fact, the idea of using xenodiagnosis to check for parasitic diseases, in particular American trypanosomiasis, was nothing more than a reinterpretation, based on his experiences in Brazil, of scientific work he had been doing since 1904, when he “studied the evolution of fish trypanosomes and observed ... the appearance of abundant cultures of parasites in the digestive tract of young leeches that had fed off the blood of fish deemed free from infection” when analyzed under a microscope (Brito, Lima, 2009, p.196).

According to Brumpt, this diagnostic method had several advantages over other ways of diagnosing Chagas disease, namely, making a direct examination of blood, making inoculations in laboratory animals, and producing cultures. For one thing, a kissing bug could absorb more blood then could be examined “between a slide and its cover” under a microscope; also, there was great familiarity with the populations affected by the kissing bug and its “painless bite;” not to mention the inconvenience of transporting a microscope on horseback to the distant regions where the disease was most common (Brumpt, 1914, p.100, 101; Kropf, 2009).

As with the other works he produced when in São Paulo, the article in which Brumpt proposed xenodiagnosis was originally published in the pages of *Anais Paulistas de Medicina e Cirurgia*. This particular article was only published in November 1914, or after his “sudden departure,” on October 4, to contribute to the French war effort (Brumpt, 1914).

Brumpt promised to return to this part of Brazil once an armistice was signed; indeed, he left his teaching materials and suitcases containing his personal effects at the faculty under the care of Celestino Bourroul (Bourroul, 2 maio 1920). Yet he was unable to fulfil his promise. On May 29, 1919, Émile Brumpt wrote to Arnaldo de Carvalho notifying him that he would not be resuming his role in São Paulo. He explained that with the sudden death of Raphael Blanchard by heart failure, all his plans had “been modified.” After the death of his mentor, he was the unanimous choice of his peers to succeed him as professor of parasitology at the Paris Faculty of Medicine.

Assuming a friendly, cordial tone, the letter goes on to say that “throughout the war, for reasons of discretion,” he had not wished to bother Carvalho by “speaking of financial questions,” but as peace was returning and the cost of life in Paris was rising after the war, he wondered whether the director would “kindly examine the compensation” he deemed fair to receive from the state of São Paulo. He claimed a total of eight thousand francs. Of this total, five thousand francs was for the practical work in parasitology done during half of 1914; one thousand was for loans made in cash for the purchase of animals and other expenses during his travels (Bauru and Lassance); and another two thousand francs was “partial compensation for the passage” of his family from Brazil to France. He argued that he was owed this sum because the contract had been terminated “for reasons of force majeure.” He therefore did not consider it necessary to call on the intervention of the Ministry of Foreign Affairs to regulate this issue. He ended the letter congratulating Carvalho on the “great success of your Faculty of Medicine” (Brumpt, 26 abr. 1919).

It would appear that this letter went unanswered, because Brumpt sent a new correspondence on January 26, 1920. Using less friendly and more direct language, he notified his correspondent that he had contacted the Ministry of Public Education to “obtain by the hierarchy” the sum claimed, which he believed the “wealthy state of São Paulo could easily” afford to pay him. In his three-page-long request to the ministry, he set forth his demands in detail and added a claim for more five thousand more francs should the material he had left in Brazil not be returned to him (Brumpt, 26 jan. 1920).

Upon receipt of this claim, the Ministry of Public Education contacted the French consul in São Paulo, who in turn replied stating that he had contacted Carvalho and that the case could be deemed closed, since he had shown him the stub of the check for eight thousand francs he had sent to Brumpt. Ask for his belongings, these were returned to France little by little, case by case, transported on the request of Bourroul by people who were traveling between São Paulo and Paris, as becomes clear from a letter he sent Brumpt in May 1920:

My dear friend and master Brumpt,

I have dispatched with a friend of mine – Senhor Dias – one of your heavy cases. As I do not have the key, I do not know if he will encounter any obstacle at customs at Cherbourg ... Did you receive the other two cases, send with my friend Figueiredo, containing your collections, preparations etc.? (Bourroul, 2 maio 1920).

## Final considerations

In the almost three decades between the imbroglio of the termination of Émile Brumpt’s contract with the São Paulo faculty and the celebrations of his scientific jubilee, in 1948, there was scant contact between the parties. Essentially, such contact as there was boiled down to his acceptance of students from São Paulo for periods of study at his laboratory in Paris and a short stay in the city on his way back from a trip to Uruguay and Paraguay, in 1924, when he had the pleasure of meeting with his “old colleagues and students from the Faculty of Medicine” (Brumpt, 1934, p.35).

In this interim, the course in São Paulo underwent profound changes. The importance of Europe waned as the United States gained influence, not least because of sizable financial investments made between 1916 and 1931 by the Rockefeller Foundation, which transformed the institution into “one of the most modern centers of medical education of the time” (Marinho, Mota, 2012, p.70; Marinho, 2013; Mota, 2005). In 1934, the Faculty of Medicine was joined together with other higher education establishments and research institutes in the state to form the new University of São Paulo (USP), which, in subsequent decades, developed and established a strong academic reputation nationally and internationally (Marinho, Mota, 2012, p.108, 109; Mota, 2005).

In this same period, Émile Brumpt consolidated his name as a global leader in parasitology. After his appointment as professor of parasitology at the Paris Faculty of Medicine, he was appointed director of the School of Malariology, founded at the University of Paris, and, by unanimous vote, president of the Society of Exotic Diseases (Société de Pathologie Exotique) (1932-1936). Also, together with Maurice Langeron and Maurice Neveu-Lemaire, he founded the journal *Annales de Parasitologie Humaine et Comparée* (1923), while also continuing to engage regularly in expeditions to the most far-flung areas of the tropical world, which had a considerable influence on cementing his professional reputation.

The mythification of Brumpt as the founder of parasitology in São Paulo began in 1948, when celebrations were held to mark his 50th year of uninterrupted scientific work. The Council of the USP Faculty of Medicine were unanimous in making him professor *honoris causa* and approving a generous donation of 50,000 cruzeiros to set up the Émile Brumpt international prize in parasitology, which every year would award the author of the best work in this field of research around the world, thereby keeping alive the memory of this researcher, who died of rocky mountain spotted fever in 1951.

To conclude, I consider it necessary to stress that this article in no way aims to detract from the trajectory of this French physician, much less deny the importance of his role in organizing the São Paulo School of Medicine. Rather, the aim is to demonstrate that, far from being the figure who disseminated parasitology in Brazil, Brumpt was somebody who was able to take advantage of his time in São Paulo to make a significant contribution to broadening the horizons of this field of medical science, which, in the early years of the twentieth century, was still taking its first steps and counting on investigations that were often conducted in collaboration with members of the local medical community.

## NOTES

1 It should be pointed out that the institutionalization of medical higher education in São Paulo was not the outcome of a harmonious, civilized, hegemonic process conducted by the state’s medical community. In fact, it involved political disputes and clashes of interests. The group responsible for founding the São Paulo School of Medicine and Surgery as a course in medicine with state control was the winner; however, it was not the first school of medicine to operate in the city. In 1911, after the Rivadávia Corrêa law had been passed, reorganizing higher education in the country and permitting the creation of private establishments, the University of São Paulo was created, which had a course in medicine and actually rivaled the state faculty until 1917, when it closed its doors. For more on this subject, see Mota (2005), Teixeira (2007), and Maia (2017).

2 Most of the literature that concerns itself with the creation of the São Paulo School of Medicine and Surgery states that the inaugural lesson was given by Edmundo Xavier (Marinho, Mota, 2012; Mota, 2005; Marinho, 2013, p.113; Fonseca, 2002). However, Silva (2003, p.177) affirms that “the opening lesson of the course was presented by the professor of natural history ... Celestino Bourroul.” This same information is in a report published on June 2, 1917, in the *Correio Paulistano* newspaper on the history of the course (O ensino..., 2 jun. 1917). This leads me to believe that the opening ceremony consisted of two (or more) lessons at the São Paulo Polytechnic School.

3 As the faculty did not have its own premises, the first lessons were given at the Alvares Penteado School of Trade and the Polytechnic. In 1914, the course was transferred to a rented building on Brigadeiro Tobias street, where it remained until 1931 (Mota, 2005; Marinho, Mota, 2012; Fonseca, 2002).

4 For further information on Arnaldo Vieira de Carvalho, see Marinho (2014); Dantes, Silva (2012).

5 Carvalho was appointed director of the course by Francisco Alves de Paula Rodrigues Alves by means of an executive order published on January 7, 1913 (Mota, 2005; Teixeira, 2007; Fonseca, 2002).

6 Article 26 of the law creating the Faculty of Medicine contains the following information: “The government is authorized to spend up to five hundred contos de réis in the execution of this law for the acquisition of material indispensable for preparing the laboratories and teaching activities, hereby opening the necessary credit” (São Paulo, 19 dez. 1912).

7 Brumpt commenced his inaugural lesson with the following: “Esteemed director, gentlemen, before I address the matters of the course we will commence, I wish to express once again my happiness, as a Frenchman, to be the first foreigner to be invited to speak at this school” (Brumpt, 21 jun. 1913, p.1). As we shall see, he only reached São Paulo in July 1914, and therefore three months after the beginning of the academic year. According to Mota (2005, p.190), the first foreign professors to arrive at the faculty were the Italian Alfonso Bovero and the Frenchman Lambert Mayer.

8 Created in 1902 after three years of negotiations between the Paris Faculty of Medicine and Union Coloniale, the Institute of Colonial Medicine had the objective of studying exotic and parasitic diseases for civil purposes. It ended up attracting many students from Latin America keen to study tropical diseases (Opinel, 2008, p.391, 392).

9 In French, Brumpt’s academic titles are: *licence ès sciences. sciences naturelles: zoologie, botanique, géologie* (1896); s*ciences physiques: chimie génerale; doctorat ès sciences naturelles* (1901), and d*octorat en medicine* (1906) (Brumpt, 1934, p.5).

10 This and other citations of texts in French and Portuguese are free translations into English.

11 For more background information on the relationships established between sleeping sickness and European imperialist projects, see Neill (2012), and Amaral (2012).

12 For a robust analysis of the trajectory of this Swiss-Brazilian physician, see the collections *Adolpho Lutz: obra completa* (12 volumes), edited by Jaime Larry Benchimol and Magali Romero de Sá, especially Benchimol (2005); Benchimol, Sá (2006).

13 On July 20, 1908, *O Paiz* newspaper reproduced in its section on the Senate Finance Committee the authorization for the payment of 4:200$ réis in gold to Celestino Bourroul for his prize trip (A Comissão..., 20 jun. 1908).

14 According to Marinho (2016, p. 116), on this trip Celestino Bourroul spent time at the Institut Pasteur in Montpellier and the Institute of Colonial Medicine, in France; the anatomical pathology laboratory at the University of Berlin; and an unspecified institution in Vienna for a specialization in medical practice, radiology, and anatomical pathology.

15 There are two boxes at the historical archives of the Institut Pasteur in Paris containing documents related to Brumpt’s missions in São Paulo in 1913 and 1914, namely BPT.D1 and BPT.D2. Specifically, they contain books, scientific articles, personal notes, field notebooks, and considerable correspondence with physicians from Brazil, above all Celestino Bourroul. There are also some separate documents bearing Bourroul’s name that suggest he may have been responsible for sending two books, written in French, on the geographical, cultural, and socioeconomic features of the state of São Paulo (Casabona, 1910; L’État..., 1911).

16 In the preface to the second edition of *Précis de Pathologie*, in 1913, written when he was in Brazil, Brumpt attributed its launch to “certain laboratory work I have been undertaking into various mammal Tripanossomos, especially *Trypanosoma cruzi*” (Brumpt, 21 jun. 1913, p.XV).

17 As we shall see, Brumpt was very interested in the leishmaniases encountered in South America. In the first edition of his treatise, he demonstrates great knowledge of studies by researchers from the region. He was also the first foreign author to validate *Leishmania braziliensis* as a specific species of this genus of protozoan. For further information on the history of leishmaniasis in a non-Eurocentric context, see Benchimol, Jogas Jr. (2020) and Benchimol, Peixoto (2022).

18 This information comes from Brumpt (1948, p.49).

19 For further information on the projects that culminated in the creation of the São Paulo School of Medicine and Surgery, see Mota (2005), Teixeira (2007), and Silva (2003).

20 Léo Lopes de Oliveira was chosen to assist the French parasitologist as a natural history assistant; however, he died in September 1913, shortly after Brumpt’s arrival (Homenagem..., 1916, s.p.).

21 No biographic information was found on this person. Silva (2003, p.166, 256) and Teixeira (2007, p.91) make reference to Synesio Rangel Pestana as a physician from Santa Casa de Misericórdia de São Paulo and a member of the São Paulo Society of Medicine and Surgery. It is possible that the surname “Rocha” was written incorrectly in the aforementioned report.

22 Details of the course in parasitology can be found in Notícias (1913, p.31-32).

23 Considered the best student of the first class, Ernesto de Souza Campos would become a histology assistant soon after graduating and then a professor, in 1925. For more details, see Maia (2017).

24 The previous meeting was held on July 15, the date on which Brumpt disembarked at Santos. At the beginning of the minutes, it is stated that “Dr. Alves Lima informed those present that the notable Professor Brumpt, who is coming as professor of natural history of the São Paulo Faulty of Medicine and Surgery, was welcomed in the name of the society” (Ata...., ago.-set. 1913, p.10).

25 For more on the issues of the São Paulo hinterlands (*sertões*) and the diseases there, see Mota (2021). On the genocide of indigenous people in this region, see Dornelles (2017).

26 For more on the research done by Chagas in his bid to understand the transmission of leishmaniases, see Chagas (1972, p.143), originally published in 1913.

## References

[B1] (1908). A COMISSÃO de finanças do Senado. O Paiz.

[B2] (1904). A DOENÇA do sono. Correio Paulistano.

[B3] AMARAL Isabel (2012). Bactéria ou parasita? A controvérsia sobre a etiologia da doença do sono e a participação portuguesa, 1898-1904. História, Ciências, Saúde – Manguinhos.

[B4] (1914). AS FAZENDAS de café e a higiene (paludismo - opilação - ferida brava). O Estado de São Paulo.

[B5] (1913). ATA da sessão ordinária realizada em 15 de junho de 1913. Arquivo da Sociedade de Medicina e Cirurgia de São Paulo.

[B6] (1913). ATA da sessão ordinária realizada em 15 de junho de 1913.

[B7] BENCHIMOL Jaime Larry, Benchimol Jaime Larry, Sá Magali Romero (2005). Adolpho Lutz, obra completa.

[B8] BENCHIMOL Jaime Larry, JOGAS Denis Guedes (2020). Uma história das leishmanioses no Novo Mundo (fins do século XIX aos anos 1960).

[B9] BENCHIMOL Jaime Larry, PEIXOTO Cláudio de Oliveira (2022). Uma história das leishmanioses no Novo Mundo: anos 1960 ao século XXI - Amazônia.

[B10] BENCHIMOL Jaime Larry, SÁ Magali Romero, Benchimol Jaime Larry, Sá Magali Romero (2006). Adolpho Lutz, obra completa.

[B11] BERTOLLI Claudio, Mota André (2021). Os sertões paulistas: medicina, saúde pública e invenção do território.

[B12] BOURROUL Celestino (1920). Correspondência de Celestino Bourroul a Émile Brumpt]. Fundo Émilie Brumpt. Missão ao Brasil. Correspondências. BPT. D1.

[B13] BOURROUL Celestino (1913). Lição de abertura do Curso de História Natural Médica. Anais Paulistas de Medicina e Cirurgia.

[B14] BRITO Constança C., LIMA Marli M, Carvalheiro José da Rocha (2009). Clássicos em doença de Chagas: histórias e perspectivas no centenário da descoberta.

[B15] BRUMPT Émile (1948). Jubilé du professeur Émile Brumpt. Fundo Émile Brumpt. Documentos biográficos. Homenagens. Jubileu BPT. A2.

[B16] BRUMPT Émile (1936). Précis de Parasitologie.

[B17] BRUMPT Émile (1934). Titres et travaux scientifiques.

[B18] BRUMPT Émile (1920). Correspondência de Émile Brumpt a Arnaldo Vieira de Carvalho.

[B19] BRUMPT Émile (1919). Correspondência de Émile Brumpt a Arnaldo Vieira de Carvalho.

[B20] BRUMPT Émile (1914). O xenodiagnóstico. Aplicação ao diagnóstico de algumas infecções parasitárias e em particular à tripanossomose de Chagas. Anais Paulistas de Medicina e Cirurgia.

[B21] BRUMPT Émile (1913). Aula inaugural. Natureza, ambiente e parasitismo.

[B22] BRUMPT Émile, DA SILVA Pirajá (1912a). Existence du "Schizotrypanum Cruzi" Chagas, 1909 à Bahia (Matta de São João) Biologie du "Conorhinus megistus". Bulletin de la Société de Pathologie Exotique.

[B23] BRUMPT Émile, DA SILVA Pirajá (1912b). Pénétration du Schizotrypanum Cruzi à travers la muquese oculaire saine. Bulletin de la Société de Pathologie Exotique.

[B24] BRUMPT Émile, GOMES João Florencio (1914). Descrição de uma nova espécie de Triatoma (T. chagasi) hospedeiro primitivo do Trypanosoma cruzi Chagas. Anais Paulistas de Medicina e Cirurgia.

[B25] BRUMPT Émile, PEDROSO Alexandrino de Moraes (1913). Pesquisas epidemiológicas sobre a Leishmaniose americana das florestas no Estado de São Paulo (Brasil). Anais Paulistas de Medicina e Cirurgia.

[B26] CAPONI Sandra (2003). Coordenadas epistemológicas de la medicina tropical. História, Ciências, Saúde - Manguinhos.

[B27] CAPONI Sandra (2002). Trópicos, micróbios y vectores. História, Ciências, Saúde - Manguinhos.

[B28] CARVALHO Arnaldo Vieira de (1913). Ofício da Diretoria da Escola de Medicina e Cirurgia de São Paulo.

[B29] CARVALHO Arnaldo (1913). Correspondência enviada a Émile Brumpt.

[B30] CARVALHO Arnaldo (1913). Telegrama a Firmiano de Morais Pinto.

[B31] CARVALHO Arnaldo Correspondência enviada a Émile Brumpt.

[B32] CASABONA Louis (1910). São Paulo du Brésil. Notes d'un colon français.

[B33] CENTRO Acadêmico "Oswaldo Cruz" (1913). Correio Paulistano.

[B34] CHAGAS Carlos, Batista Djalma da Cunha (1972). Sobre o saneamento da Amazônia.

[B35] CONFERÊNCIA Científica no Instituto Histórico (1913). A úlcera de Bauru - os resultados obtidos pelo professor Brumpt. Correio Paulistano.

[B36] DANTES Maria Amélia Mascarenhas, SILVA Márcia Regina Barros da (2012). Arnaldo Vieira de Carvalho e a história da medicina paulista (1867-1920).

[B37] DORNELLES Soraia Sales (2017). A questão indígena e o Império: índios, terra, trabalho e violência na província paulista, 1845-1891.

[B38] FACULDADE de Medicina (1913). Recepção do professor Brumpt.

[B39] FONSECA Maria Rachel Froes de (2002). Faculdade de Medicina e Cirurgia de São Paulo [verbete]. Dicionário Histórico-Biográfico das Ciências da Saúde no Brasil (1832-1930).

[B40] (1916). HOMENAGEM ao Dr. Léo Lopes de Oliveira. Revista de Medicina.

[B41] HÓSPEDES e viajantes. Professor Brumpt.

[B42] (1914). IMPALUDISMO. Correio Paulistano.

[B43] JOGAS Denis Guedes (2022). The South American medical communities in the genesis of the tropical medicine: construction and circulation of knowledge on American leishmaniasis in the beginning of the twentieth century. Medical History.

[B44] JOGAS Denis Guedes (2017). Trópicos, ciência e leishmanioses: uma análise sobre circulação de saberes e assimetrias. História, Ciências, Saúde - Manguinhos.

[B45] KROPF Simone (2009). Doença de Chagas, doença do Brasil: ciência, saúde e nação (1909-1962).

[B46] (1911). L'ÉTAT de São Paulo. Renseigments utiles.

[B47] MAIA Ana Beatriz Feltran (2017). O apostolado de Ernesto de Souza Campos: modelos, projetos e espaços universitários (1900-1937).

[B48] MARINHO Maria Gabriela (2016). A Fundação Rockefeller e a medicina tropical em São Paulo. Circuitos, redes e personagens da parasitologia médica, microbiologia e anatomia patológica (1918-1969). Anais do Instituto de Higiene e Medicina Tropical.

[B49] MARINHO Maria Gabriela, Marinho Maria Gabriela S.M.C., Mota André (2014). Medicina, saúde e história: textos escolhidos e outros ensaios.

[B50] MARINHO Maria Gabriela, Marinho Gabriela, Mota André (2013). Caminhos e trajetos da filantropia científica em São Paulo: a Fundação Rockefeller e suas articulações no ensino, pesquisa e assistência para a medicina e saúde (1916-1952).

[B51] MARINHO Maria Gabriela, MOTA André (2012). Da Faculdade de Medicina e Cirurgia de São Paulo à Faculdade de Medicina da Universidade de São Paulo: conjunturas e contextos.

[B52] MOTA André (2021). Os sertões paulistas: medicina, saúde pública e a invenção do território.

[B53] MOTA André (2005). Tropeços da medicina bandeirante: medicina paulista entre 1892-1920.

[B54] NEILL Deborah (2012). Networks in tropical medicine: internationalism, colonialism, and the rise of a medical specialty, 1890-1930.

[B55] (1913). NOTAS. Correio Paulistano.

[B56] (1913). NOTAS. Correio Paulistano.

[B57] (1913). NOTAS. Correio Paulistano.

[B58] (1913). NOTÍCIAS. Anais Paulistas de Medicina e Cirurgia.

[B59] (1917). O ENSINO em S. Paulo. A Faculdade de Medicina e Cirurgia. Correio Paulista.

[B60] OPINEL Annick (2008). The emergence of French Medical Entomology: the influence of universities, the Institut Pasteur and military physicians (1890-c.1938). Medical History.

[B61] OPINEL Annick, GACHELIN Gabriel (2005). Émile Brumpt's contribution to the characterization of parasitic diseases in Brazil, 1909-1914. Parassitologia.

[B62] OSBORNE Michael (2014). The emergence of tropical medicine in France.

[B63] PINTO Firmiano (1913). Carta a Arnaldo de Carvalho.

[B64] RIBEIRO Maria Alice Rosa (1996). Lições para a história das ciências no Brasil: Instituto Pasteur de São Paulo. História, Ciências, Saúde - Manguinhos.

[B65] SÃO PAULO (estado) (1912). Lei n.1357, de 19 de dezembro 1912. Estabelece o curso da Escola de Medicina e Cirurgia de São Paulo, criado pela lei n.19, de 12 novembro de 1891, e dá outras providências.

[B66] SILVA Marcia Regina Barros da (2003). O mundo transformado em laboratório: ensino médico e produção de conhecimento em São Paulo de 1891 a 1933.

[B67] SOUZA CAMPOS Ernesto de (1948). Professor Brumpt. A Gazeta.

[B68] SOUZA CAMPOS Ernesto de (1913). Carta de agradecimento do Centro Acadêmico "Oswaldo Cruz" ao professor Émile Brumpt.

[B69] TEIXEIRA Luiz Antonio (2007). Na arena do Esculápio: a Sociedade de Medicina e Cirurgia de São Paulo (1895-1913).

[B70] TEIXEIRA Luiz Antonio (1995). Ciência e saúde na terra dos bandeirantes: a trajetória do Instituto Pasteur de São Paulo no período de 1903-1916.

