# Supplementary material for: The institutionalization of parasitology in São Paulo: the contributions of Émile Brumpt between 1913 and 1948
Source: Hist Cienc Saude Manguinhos. 2024 Oct 11;31:e2024042. doi: 10.1590/S0104-59702024000100042en (PMC11472783; doi:10.1590/S0104-59702024000100042en)
Supplement: Translation

# Translation: A institucionalização da parasitologia em São Paulo: a atuação de Émile Brumpt no período 1913-1948

**DOI:** 10.1590/S0104-59702024000100042

**Authors:** Denis Guedes Jogas

**Affiliations:** i Pesquisador em estado pós-doutoral, Departamento de Pesquisa/Casa de Oswaldo Cruz/Fiocruz. Rio de Janeiro – RJ – Brasil denis.jogas@hotmail.com

**Keywords:** Émile Brumpt (1877-1951, Escola de Medicina e Cirurgia de São Paulo, Parasitologia, Leishmaniose, doença de Chagas

## Abstract

O artigo, por meio de volumosa documentação coletada no Arquivo Histórico do Instituto Pasteur de Paris, na Biblioteca de Manguinhos da Fundação Oswaldo Cruz e na Hemeroteca Digital da Biblioteca Nacional do Brasil, analisa as relações estabelecidas entre a institucionalização da parasitologia em São Paulo e a presença do médico francês Émile Brumpt nos anos iniciais da Escola de Medicina e Cirurgia de São Paulo. Utilizando fontes primárias inéditas ou pouco conhecidas, este artigo pretende contribuir tanto para a historiografia que versa sobre as relações intelectuais franco-brasileiras como também para a memória institucional da atual Faculdade de Medicina da Universidade de São Paulo.

Brumpt, ajudante [de] Blanchard quer lecionar história natural na nova faculdade, condições? Procure também professor idôneo [para] clínica médica responda urgente, pagarei despesas. Arnaldo (Carvalho, 27 jan. 1913).

Em 2 de abril de 1913, um dos maiores e mais antigos anseios de expressiva (e politicamente influente) parte da classe médica paulista começou a ganhar forma e real concretude.^1^ Com a promulgação da lei estadual 1.357 de 19 de dezembro de 1912 e sua posterior regulamentação pelo decreto 2.344 de 31 de janeiro de 1913, iniciaram-se as atividades do curso preliminar da Escola de Medicina e Cirurgia de São Paulo, com as aulas inaugurais de física médica ministrada por Edmundo Xavier (1861-1933) e de história natural por Celestino Bourroul (1880-1958) no anfiteatro da Escola Politécnica de São Paulo.^2^

A despeito das limitadas e improvisadas instalações físicas,^3^ Arnaldo Augusto Vieira de Carvalho (1867-1920),^4^ influente médico paulista que foi escolhido como primeiro diretor da escola,^5^ ficou encarregado da organização e funcionamento do novo curso médico (Mota, 2005; Fonseca, 2002; Marinho, Mota, 2012). Por meio de prerrogativas descritas na lei e decreto acima mencionados, buscou formar robusto corpo docente composto por experientes médicos nacionais e estrangeiros, além de adquirir “material indispensável à montagem de laboratórios”, muitas vezes, comprado no exterior.^6^

O primeiro professor estrangeiro a ser contratado foi Alexandre Joseph Émile Brumpt (1877-1951) para reger a cadeira de história natural.^7^ Principal discípulo de Rafael Blanchard, fundador do Instituto de Medicina Colonial de Paris,^8^ Brumpt aos 36 anos já era considerado um renomado médico francês quando esse convite foi feito. Além de suas experiências docentes como “preparador”, “chefe de trabalhos práticos” e “professor associado” [*professeur agrégé*] na Faculdade de Medicina de Paris, ele já havia comandado expedições científicas ao mundo tropical e publicado em 1910 a primeira edição de seu manual *Précis de Parasitologie*, que contaria com uma série de reedições nos anos seguintes (1913, 1922, 1927, 1936, 1949) e muito influenciou os médicos não apenas de São Paulo, como também de toda a região latino-americana.

A contratação do parasitologista francês foi considerada um grande triunfo da nascente instituição que, naquele momento, rivalizava sua própria existência com outra recém-fundada faculdade de medicina, de caráter privado, na cidade de São Paulo. A vinda de renomados professores estrangeiros pode ser vista como uma provável estratégia de Carvalho e seu grupo para derrotar o curso concorrente (Mota, 2005; Teixeira, 2007). Como veremos ao longo deste texto, a participação de Brumpt nos anos iniciais do curso médico estatal teve tal repercussão, que, *a posteriori*, ele foi lembrado pela memória institucional como fundador desse campo médico em São Paulo, apesar da sua breve atuação como professor desse curso médico e dos significativos embates ocorridos com parte do corpo discente.

Neste artigo, pretendo explorar alguns momentos dessa história, como a trajetória pregressa de Émile Brumpt, as circunstâncias de sua contratação, principais atividades realizadas durante a estada em São Paulo, os meandros de seu desligamento após eclosão da Primeira Guerra Mundial e, por último, a ativa participação da Congregação da Faculdade de Medicina nas comemorações do jubileu científico desse pesquisador, ocorridas em 1948, em Paris.

## Conexões São Paulo-Paris: contatos e intercâmbios impulsionados por tripanossomíases humanas e outras doenças parasitárias

A epígrafe com a qual iniciei este artigo é um trecho do telegrama que Arnaldo Vieira de Carvalho enviou a Firmiano de Morais Pinto (1861-1938), comissário do estado de São Paulo para França e Suíça, informando que o “discípulo [de] Blanchard” havia aceitado o convite para assumir a cadeira de história natural na nova escola de medicina de São Paulo. Nele, Carvalho solicitava ao comissário que averiguasse, com urgência, as condições exigidas pelo parasitologista. Em 5 de fevereiro, Pinto respondeu detalhando o pedido de Brumpt: “30 mil francos anuais, contrato de 2 anos escolares e 4 passagens de ida e volta” no trajeto Paris-São Paulo. Na carta, o comissário ainda informava que, caso o contrato fosse firmado, o francês estaria disponível para assumir seu posto em São Paulo em junho do ano corrente e, assim como fizera Carvalho, solicitava uma resposta urgente do médico paulista (Pinto, 5 fev. 1913).

Formado em ciências naturais com ênfase em zoologia, botânica e geologia (1896), ciências físicas com ênfase em química geral (1897) e títulos de doutor em ciências naturais (1901) e medicina (1906),^9^ Émile Brumpt ganhou projeção nos círculos médicos franceses e estrangeiros após realizar duas viagens ao continente africano. A primeira teve dimensões e características de uma verdadeira epopeia humana com duração de 27 meses (janeiro de 1901 a março de 1903). Na qualidade de médico e naturalista da missão comandada pelo visconde de Bourg do Bozas (que, acometido por malária e exaustão, falecera durante o percurso), cruzou a África equatorial adentrando o continente por Djibouti, no mar Vermelho, até chegar a Braziville (atual capital da República do Congo), quando saiu pelo oceano Atlântico. Durante quase todo o percurso, ele utilizou apenas uma bússola para localização e guiamento, e aproveitou a ocasião para recolher abundante material científico que lhe permitiu “organizar os trabalhos práticos de parasitologia da Faculdade de Medicina, assim como aqueles do Instituto de Medicina Colonial”^10^ (Brumpt, 1934, p.30).

A segunda expedição, realizada apenas quatro meses após seu retorno a Paris, foi ao Congo Francês, teve duração de quatro meses (julho a novembro de 1903) e o objetivo de verificar a hipótese emitida durante a primeira expedição de que as “as moscas tsé-tsé, frequentemente [encontradas] nas bordas dos rios, eram o agente vetor da doença do sono” (Brumpt, 1934, p.30). Ao retornar à Europa, Brumpt desembarcou na França acompanhado de Bobangui, Makaya e Salomão, três colonos congoleses que padeciam da tripanossomíase africana (doença do sono) e no Hôspital des Dames Française serviram de objeto de estudo para os médicos europeus, antes de falecerem um após o outro.^11^ A notícia da existência de humanos acometidos por essa doença motivou visita a Paris de comitiva da London School of Tropical Medicine formada por diversos próceres da medicina tropical inglesa como Patrick Manson, James Cantile, George Low e Louis Sambon (Osborne, 2014, p.207, 208; A doença..., 20 fev. 1904).

É importante lembrar que o mundo médico do início do século XX é marcado por significativas alterações na compreensão da causalidade e natureza das doenças infectocontagiosas, decorrentes, sobretudo, da revolução microbiana, da doutrina do agente etiológico único e individualizado de cada manifestação mórbida, e, em especial, da teoria dos mosquitos, que postulara a possibilidade de diferentes espécies de insetos hematófagos serem capazes de não apenas transmitir como também atuar como hospedeiros intermediários, nos quais agentes patógenos aos seres humanos e animais desempenhavam importantes fases de seus ciclos evolutivos. Essas alterações na semiótica das doenças dinamizaram as relações entre áreas da história natural, como a entomologia e a zoologia, e as pesquisas biomédicas ao fornecer um princípio e/ou modelo para que pesquisadores espalhados por diferentes regiões buscassem responder aos desafios de construção do quebra-cabeça chamado de “doenças tropicais”, isto é, relacionar manifestações clínicas com seus respectivos vetores e agentes patogênicos, em especial, quando se tratava de territórios com climas quentes e úmidos (Benchimol, Sá, 2006; Benchimol, Jogas Jr., 2020; Jogas Jr., 2017, 2022; Caponi, 2002, 2003).

Mais ou menos ao mesmo tempo que Brumpt ganhava projeções por suas pesquisas sobre a doença do sono, o então estudante Celestino Bourroul defendeu tese intitulada *Mosquitos do Brasil* na Faculdade de Medicina da Bahia. Com orientação de Adolpho Lutz,^12^ seu trabalho foi realizado a partir de pesquisas entomológicas feitas na ilha de Itaparica. Agraciado com a láurea acadêmica, recebeu como premiação viagem à Europa para complementação de sua formação (Benchimol, Sá, 2006, p.58; Marinho, 2016, p.116). Após demorados trâmites burocráticos, o pagamento do crédito foi autorizado pelo Senado Federal apenas no segundo semestre de 1908,^13^ permitindo-lhe transitar por diferentes instituições científicas de França, Alemanha e Áustria, dentre as quais destaca-se, para o objetivo deste trabalho, o próprio Instituto de Medicina Colonial.^14^

A passagem de Bourroul pela Cidade Luz, antes de seu retorno a São Paulo (em meados de 1910), foi o marco inicial de uma longeva relação estabelecida com Émile Brumpt, tornando-o principal interlocutor do francês em São Paulo, tanto durante sua estada na cidade quanto após seu retorno à França na resolução das questões burocráticas relacionadas ao seu desligamento que serão esmiuçadas na sequência deste texto.^15^ Além disso, como já mencionado, Celestino Bourroul foi o responsável por iniciar e conduzir o curso de história natural desde o início do ano letivo, em 15 de abril, até a chegada do professor francês, em julho daquele ano, um mês, portanto, após a data previamente informada pelo comissário (Bourroul, 1913, p.1-13).

Carvalho reconhecia na contratação de Brumpt um importante evento da fase inaugural do novo curso médico e, por isso, esforçou-se para atingir as condições ideais para execução do trabalho docente, além de conceder grande liberdade na organização do seu programa de curso, como ficou claro nas primeiras correspondências enviadas ao parasitologista francês:

Querido e honrado colega,

Eu acabo de receber a cópia das correspondências trocadas entre você e o comissário do Estado em Paris e agora estou certo de ter sua colaboração na organização da nova escola de medicina de São Paulo. Eu comuniquei suas últimas condições ao secretário do interior e acredito que ele não terá nenhuma dificuldade em aceitá-las ...

Eu estou muito contente de poder contar contigo para começar bem o ensino das ciências médicas na nossa nova escola e, junto comigo, todo o mundo médico de São Paulo se alegra de poder receber as lições de um sábio cujo nome é bem conhecido por eles há muito tempo ...

Você vem para uma escola que começa e cuja organização do ensino da cadeira de ciências naturais (principalmente parasitologia) vos está reservado. Eu lhe darei algumas explicações sobre a acepção que eu dou a este título – ciências naturais (principalmente parasitologia).

O estudo das ciências naturais (botânica, zoologia) é feito ou deve ser feito no liceu. Nós não temos nada a ver se não com as partes dessa disciplina em relação direta com a medicina, então, com a parasitologia animal e vegetal.

Dessa forma, no curso, o professor não irá se ocupar da botânica e nem da zoologia geral. Ele fará tão simplesmente algumas lições no começo, para demonstrar as relações da parasitologia com a botânica e zoologia e entrará, em seguida, no estudo especial dos parasitas. A distribuição dos estudos, o método, a seriação das matérias, tudo isso será deixado a vosso julgamento e eu creio que sendo um professor experiente como você, não há nada melhor a fazer. Entretanto, os trabalhos devem começar no mês de abril e neste momento você ainda estará ausente. A sua espera, eu organizei um programa baseado em vosso tratado de parasitologia [*Précis de Parasitologie*], que poderá ser modificado após sua chegada.

Aqui em São Paulo nós não temos a nossa disposição todo o necessário para o ensino de vossa cadeira. Por isso, seria apropriado para escola, vos encarregar de fazer a aquisição, na França, dos objetos indispensáveis. Você poderia se encarregar disso? Em caso afirmativo, tenha a boa vontade de fazer um cálculo da importância necessária e me comunicar. Eu obterei rapidamente a autorização do governo ... e encarregarei meu correspondente ... de vos facilitar a compra e a expedição de todas essas coisas (Carvalho, s.d.).

Brumpt, por sua vez, respondeu ao diretor enumerando o material científico que considerava necessário adquirir, pedindo alguns detalhes adicionais sobre o correspondente da faculdade em Paris, averiguando a possibilidade de um retorno a sua terra natal em outubro do mesmo ano e perguntando sobre a existência de remuneração adicional pela condução de trabalhos práticos complementares em parasitologia. Em carta datada de 14 de abril de 1913, Carvalho respondeu deixando-o absolutamente à vontade para comprar o material que julgasse necessário, escolher o correspondente que melhor lhe conviesse em Paris e explicando que “o tempo do seu trabalho vai de março a junho e de julho a novembro”, sendo, portanto, “impossível retornar a França em outubro”, mas ressaltava que ele poderia ir “em novembro, após os exames”. Assim, teria “os meses de dezembro, janeiro, fevereiro e março para ajustar seus afazeres e retornar, em seguida, aos seus trabalhos aqui [em São Paulo]”. Em relação aos trabalhos práticos complementares, dizia ser impossível prometer qualquer coisa, pois não tinha previsão orçamentária para esse tipo de trabalho. “Em todo caso”, prosseguia, “traga suas preparações e eu vos prometo de empregar todos os esforços para vos ser agradável, pois eu realmente quero ver você entre nós por causa da nossa escola e dos muitos problemas de nossa patologia tropical” (Carvalho, 14 abr. 1913).

E, de fato, Carvalho cumpriu o prometido. Em ofício da diretoria da Escola datado de 18 de setembro de 1913, portanto, após a chegada do médico francês ao Brasil, vinha escrito:

De acordo com vossa solicitação e com autorização do Snr. Dr. Secretário do Interior, comunico que vos é concedido alocação de sete mil francos no ano de 1913 e dez mil francos no ano de 1914 pela direção de trabalhos práticos do Laboratório de Parasitologia. Esse suplemento vos será pago ao terminar o ano letivo, isto é, em novembro dos respectivos anos (Carvalho, 18 set. 1913).

Neste momento da narrativa, uma pergunta se impõe: por que Émile Brumpt aceitou o convite para vir ao Brasil? Não acredito ter sido pelo salário que o governo de São Paulo aceitou pagar e muito menos pela amizade nutrida com Celestino Bourroul, mas, sim, por aspirações profissionais relacionadas, sobretudo, a duas constatações – ou descobertas – científicas recentemente realizadas por membros da comunidade científica nacional. A primeira foi a descrição do *Trypanosoma cruzi* e da tripanossomíase americana (doença de Chagas), única outra tripanossomíase humana verificada no mundo, além da doença do sono (tripanossomíase africana), objeto de estudo a que o francês se dedicava há bastante tempo.^16^ A segunda foi a identificação de protozoários do gênero *Leishmania* pela primeira vez no hemisfério ocidental na cidade de Bauru, região Noroeste de São Paulo, e a subsequente controvérsia sobre se tratar de uma espécie de protozoário desse gênero diferenciada da América do Sul, que, por sua vez, seria responsável por uma doença igualmente particularizada dessa região: a leishmaniose tegumentar – ou florestal – americana.^17^

Além disso, durante discurso proferido por ocasião do jubileu científico, em 1948, Brumpt reconheceu como um importante personagem no processo decisório para aceitar o convite do governo de São Paulo, Carlos José Botelho Jr., que, naquele momento, era estudante de medicina na capital francesa.^18^ Neto de Antônio Carlos de Arruda Botelho, barão, visconde e, posteriormente, conde de Pinhal, Botelho Jr. fazia parte de uma influente família da política paulistana. Ao cursar medicina em Paris, seguiu os passos de seu pai, Carlos José de Arruda Botelho, que, em 1878, lá se formara e após retornar a São Paulo tornou-se médico da Santa Casa de Misericórdia e ativo partícipe nos distintos projetos que culminaram, em 1913, na criação da Escola de Medicina e Cirurgia de São Paulo.^19^ Ademais, ao realizar busca perfunctória sobre a genealogia de Botelho Jr., a fim de situar historicamente o personagem citado por Brumpt, descobri que sua tia Cândida de Arruda Botelho – filha do segundo casamento do seu avô – era esposa de Firmiano de Moraes Pinto. Dessa forma, é possível conjecturar que Botelho Jr. tenha sido aluno do parasitologista francês e exercido uma espécie do que hoje chamaríamos de *lobby* para convencê-lo a aceitar o convite para compor os quadros da nova escola médica de São Paulo.

## Émile Brumpt na Escola de Medicina e Cirurgia de São Paulo: pesquisa, ensino e, por fim, ruptura?

Em 15 de julho de 1913, Émile Brumpt desembarcou junto com sua esposa, Renée, do vapor Frísia, no porto de Santos. Lá, foi recebido por Celestino Bourroul e Léo Lopes de Oliveira,^20^ como representantes da faculdade, e “comissão de acadêmicos ... que o acompanharam até a capital”. Às 18h58, o trem que trouxe Brumpt chegou à Estação da Luz, na região central de São Paulo, onde o esperavam diversos políticos do estado e destacados membros da classe médica paulista como Arnaldo Viera de Carvalho, Edmundo Xavier e Antônio Carini, além de grande comitiva formada por seus futuros alunos que promoveram uma “ovação entusiasta” (Faculdade..., 16 jun. 1913).

Após o restabelecimento do silêncio, Synesio Rocha^21^ pronunciou, em francês, elogiosa saudação dando-lhe as boas-vindas “em nome da mocidade da Faculdade de Medicina e Cirurgia de S. Paulo” que estava cheia de entusiasmo por “receber grande cientista que a França inteira admirava – o mais lídimo representante da intelectualidade desse país”. O francês, por sua vez, agradeceu em poucas palavras e disse desejar “corresponder do melhor modo ao convite honroso que lhe fez o governo de S. Paulo”, antes de se dirigir ao hotel Rôtisserie, onde ficou hospedado (Faculdade..., 16 jun. 1913; Hóspedes..., s.d.).

Em 19 de julho de 1913, portanto quatro dias após o desembarque, Émile Brumpt realizou, em sessão aberta ao público, aula inaugural do curso que passaria a ministrar às quintas-feiras e aos sábados, a partir das 7h30 da manhã nas dependências da Escola de Comércio Alvares Penteado (Notícias, 1913, p.1). Como Celestino Bourroul havia conduzido o conteúdo programático até a temática dos protozoários, informou que prosseguiria a partir do estudo dos artrópodes. Na lição inaugural, entretanto, dissertou de maneira mais abrangente sobre as relações estabelecidas entre “natureza, ambiente e parasitismo ... tal como ela se apresenta sob o ponto de vista dos animais e vegetais que parasitam o homem”. Postulou que, salvo algumas exceções, como a sífilis hereditária, “os homens e animais geralmente vêm ao mundo desprovidos de infeções”, retirando da natureza e do ambiente “todos os germes que, afetando-o, o tornam doente” (Brumpt, 21 jun. 1913, p.1). Para o ano letivo, planejou ministrar “curso mais ou menos completo de parasitologia” por considerá-lo “indispensável” para os futuros médicos em razão da “relação íntima [existente] entre a medicina humana e a medicina veterinária” (p.3).^22^

O caráter prático e experimental inerente à prática e ao ensino da parasitologia ia ao encontro dos valores professados por Carvalho na constituição das bases da nova faculdade quando “procurou conferir base científica e experimental ao ensino, com ênfase na pesquisa e em práticas laboratoriais” (Marinho, Mota, 2012, p.20). Outra inovação observada foram os critérios mais flexíveis de admissão: além de aceitar alunos de ambos os sexos e destinar 10% de suas vagas para matrícula de alunos pobres, o decreto de criação da escola previa uma série de possibilidades para o ingresso direto no curso preliminar, como a “portadores de diplomas conferidos pelos ginásios oficiais”, “formados por qualquer escola superior de oficiais da República ou Estado” ou ainda para aqueles que solicitassem transferências das faculdades de medicina nacionais (Rio de Janeiro e Salvador) ou mesmo estrangeiras (Mota, 2005; Teixeira, 2007; Maia, 2017).

Tais facilidades, entretanto, não impediram e até mesmo incentivaram um rigoroso regime de ensino, uma vez que a condição única de acesso ao curso geral era a plena aprovação nas disciplinas do curso preliminar (Mota, 2005). Era, inclusive, necessário ao alunato compreender complexas lições em diferentes línguas estrangeiras, como as de parasitologia ministradas em francês e as de anatomia, conduzidas em italiano por Alfonso Bovero, proveniente da Universidade de Turim (Souza Campos, 17 mar. 1948).

Como analisado por Mota (2005), ainda durante o primeiro ano de funcionamento do novo curso médico, o sistema de avaliação por notas e a alta exigência do curso preliminar geraram protestos por parte do corpo discente de tal magnitude que levaram o diretor a ordenar o fechamento da escola entre os dias 16 e 21 de agosto. De acordo com reportagem publicada em 17 de agosto de 1913 no *Correio Paulistano*, esses alunos “descontentes com as notas baixas ... procuraram demonstrar seu desagrado” aos professores Edmundo Xavier e Émile Brumpt por meio de vaias durante suas lições. Conseguiram colocar o plano em prática durante aula do primeiro docente e pretendiam repeti-lo no curso de parasitologia. Como, “entretanto, o sr. dr. Brumpt reagisse logo, por tratar-se de um hóspede, os alunos não reproduziram a sua demonstração de desagravo”. Além das vaias, o grupo ainda partiu para ação direta ao se dirigir à clínica particular de Carvalho com intensão de depredá-la. Ao tomar ciência do ocorrido, Carvalho “tomou imediatamente as providências que o caso requeria” e afirmou que tinha apoio do governo para “agir com rigor contra os culpados”, iniciando, durante a paralisação do curso, um inquérito para averiguar os culpados (Notas, 17 ago. 1913). Logo percebeu, no entanto, que essa seria tarefa nada fácil, devido às “dúvidas quanto aos verdadeiros responsáveis pelos acontecimentos, e também o sobrenome que ostentavam muitos daqueles alunos” (Mota, 2005, p.197).

Diante disso, decidiu-se não punir nenhum aluno e reabrir as portas do curso médico em 23 de agosto; entretanto,

o retorno às aulas trouxe novas vaias aos professores Brumpt e Xavier, o que levou a diretoria, já atordoada, a fechar [novamente] a Faculdade e a convocar a Congregação, que, a 3 de setembro, de acordo com o artigo 210 do regulamento, suspendeu por um ano todos os alunos presentes à aula em que se tinham dado as perturbações. Puniram-se 95 alunos, dos quais 59 recorreram à Congregação. No dia 16, os resultados das provas parciais geraram novos conflitos em sala com Dr. Xavier e mais 34 suspensões, bem como a proibição de entrada aos alunos. Àquela altura, alguns deles, considerados pelo Dr. Arnaldo os ‘melhores da Faculdade’, que não participaram dos acontecimentos, apresentaram-se à diretoria para repudiar os atos ocorridos e colocar-se ao lado da instituição (Mota, 2005, p.196-197).

Essas desavenças resultaram em uma significativa redução do quadro discente da instituição. Dos 180 alunos que haviam se matriculado na primeira turma, apenas 70 prestaram os exames finais, dos quais 34 foram promovidos ao curso geral e os outros 36, reprovados. Além disso, essa crise teve forte repercussão no seio do Centro Acadêmico Oswaldo Cruz (Caoc). Fundado em meados de 1913, “contou [inicialmente] com alunos que fizeram oposição às posturas dos professores e da direção, sendo, por isso, desligados da faculdade e, consequentemente, do Caoc” (Mota, 2005, p.198). A partir desses acontecimentos, a direção trouxe o centro acadêmico para sua órbita de influência, e, reorganizado, sob presidência de Ernesto de Souza Campos,^23^ “ganhou a missão de colaborar com [a] diretoria e os desígnios imputados pela escola” (Mota, 2005, p.199; Maia, 2017).

Apesar das desavenças com alunos, a presença de Brumpt na cidade animou a elite médica paulista. Na sessão da Sociedade de Medicina e Cirurgia de São Paulo, realizada em 1º de agosto de 1913, primeira reunião ocorrida após sua chegada,^24^ ele foi aclamado como sócio correspondente por sugestão do médico italiano Antonio Carini, diretor do Instituto Pasteur de São Paulo havia cerca de sete anos e um dos sócios mais ativos na sociedade nesse período (Teixeira, 1995, 2007; Ribeiro, 1996). No encontro seguinte, ocorrido no dia 15 do mesmo mês, foi-lhe concedida a palavra para realizar conferência sobre higiene e parasitologia que teve duração de 1 hora e 10 minutos (Ata..., jun.-jul. 1913, p.4 e 8).

Além da participação nas sessões dessa sociedade, o médico francês buscou estabelecer parcerias com membros da comunidade médica local que ultrapassaram as meras formalidades. Dentre suas mais significativas atividades em colaboração com médicos paulistas destacam-se as incursões médico-sanitárias realizadas ao interior do estado, as quais, além dos interesses científicos pelos objetos de pesquisa em voga (como leishmaniose e doença de Chagas), estavam circunscritas em um pretenso projeto civilizatório das forças políticas estatais que, pelo avanço da cafeicultura, buscavam ocupar e afirmar a presença do Estado nos chamados sertões paulistas, mesmo que, para isso, fosse necessário promover genocídios indígenas, como aqueles ocorridos no chamado Oeste Paulista; regiões que eram propagadas pelas elites políticas como uma espécie de vazio territorial (Mota, 2021; Bertolli Filho, 2021).^25^

Foi o caso do contato com Alexandrino de Moraes Pedroso (1881-1922): formado na Universidade da Pensilvânia, era chefe do laboratório anatomopatológico da Santa Casa de Misericórdia e professor de histologia e microbiologia na mesma escola em que o francês atuava. Ao que tudo indica, além de Celestino Bourroul, foi com Pedroso que Brumpt estabeleceu os laços profissionais mais profícuos durante sua primeira estada no Brasil.

Em setembro de 1913, Émile Brumpt e Alexandrino Pedroso deram início ao que deveria ser um amplo inquérito epidemiológico sobre as leishmanioses encontradas nessa região, com realização de expedição ao interior paulista e savanas de Mato Grosso, ocorrida entre os dias 9 e 30 do referido mês, que deveria se constituir apenas como “o início de nossas pesquisas sobre a Leishmaniose americana” (Brumpt, Pedroso, 1913, p.100). A ênfase recaía em cinco aspectos da doença: (1) estudo dos casos típicos (data da aparição da doença, localização das úlceras no corpo do doente, opinião dos doentes sobre a origem de suas úlceras); (2) pesquisas sobre vetores (frequência sazonal, localização, pluralidade de picadas etc.); (3) pesquisa do ou dos reservatórios do vírus; (4) animais “inoculáveis”; e (5) experiências a instituir de futuro para resolver o problema etiológico (p.100, 101).

Durante a viagem, encontraram 65 casos, dos quais qualificaram 90% como benignos, muito semelhantes ao botão do oriente (leishmaniose cutânea). Nos 10% restantes, no entanto, a doença apresentava formas malignas, com especial tendência a se propagar para partes mucosas do corpo, o que lhes permitia considerá-la “uma afecção bem individualizada”. Suas características clínicas e epidemiológicas diferenciadas levaram os pesquisadores a denominá-la “leishmaniose florestal americana”, uma vez que, ao contrário do botão do oriente, endêmico em cidades com grandes aglomerações populacionais, grassava em áreas isoladas, com poucas habitações e próximas a matas (Brumpt, Pedroso, 1913, p.97).

Em busca dos possíveis reservatórios, examinaram diversos animais silvestres e domésticos, como anta, veado, cotia, cão, cavalo, mula, burro, boi, carneiro, cabra, gato e porco. Por outro lado, lamentaram não terem obtido sucesso em capturar “cães do mato – de difícil caça e pouco conhecidos, mas, certamente, sensíveis ao vírus que produz as úlceras”. Esperavam ter mais sucesso em “futura viagem às zonas assoladas”. Em duas ocasiões, consideraram suspeitas úlceras encontradas em cotias, mas a impossibilidade de verificar a existência de *Leishmania* nessas feridas os impediu de confirmar essa hipótese, apesar de julgarem possível emitir opinião de que “se existirem reservatório, como acreditamos, eles são constituídos por animais selvagens – sobretudo os representantes dos gêneros *Canis* e *Aguti* [cotia]” (Brumpt, Pedroso, 1913, p.129, 130).

Outra importante temática que procuravam determinar dizia respeito aos possíveis transmissores da leishmaniose florestal americana. Para isso, coletaram e examinaram diversas espécies de vermes, sanguessugas, ácaros, carrapatos, piolhos, percevejos, barbeiros, mosquitos, moscas, borrachudos (simulídeos), maruins (tabanídeos) e biriguis (flebotomíneos) que encontravam pelo caminho. Ao tentar fazer correlações epidemiológicas, no entanto, logo perceberam que essa não era uma tarefa fácil, pois “muito habituados à vida na floresta, [os doentes] pouca atenção prestam, em geral, aos insetos sugadores – com os quais, aliás, eles já se acostumaram”, tornando “impossível, mesmo para os mais conscienciosos, precisar com exatidão o animal que os pôde picar e infectar no lugar que as úlceras se reproduziram” (Brumpt, Pedroso, 1913, p.102).

Eles procuravam um animal capaz de inocular o parasito em diversos pontos do corpo humano devido aos frequentes casos de úlceras múltiplas num único indivíduo e que apresentasse hábitos diurnos, pois partiam do pressuposto de que a doença era contraída durante a jornada de trabalho nas florestas. Após elucidar os motivos pelos quais descartaram um após outro representante da vasta fauna investigada, elegeram as mutucas (tabanídeos) como possíveis transmissores da doença florestal. Suas conclusões foram baseadas em seus hábitos crepusculares, na rapidez de seu voo e na tenacidade ao picar, em especial, a cabeça e os membros de humanos e animais (Brumpt, Pedroso, 1913, p.126, 127).

Os flebótomos, que a partir da década de 1920 passariam a ser associados à transmissão das diferentes formas clínicas das leishmanioses, foram apontados por alguns doentes como os “animais mais temíveis da floresta”, devido a suas repetidas e dolorosas picadas. Seus hábitos notavelmente noturnos e recentes conclusões publicadas por Carlos Chagas após viagem ao vale do Amazonas^26^ os levaram a descartar essas moscas hematófagas como vetores da doença florestal (Brumpt, Pedroso, 1913, p.128).

No dia em que retornou à capital paulista, Émile Brumpt foi convidado por Ernesto de Souza Campos, presidente do Centro Acadêmico Oswaldo Cruz, a fazer uma conferência na sede do Instituto Histórico e Geográfico, cujo título escolhido foi “Investigações parasitológicas em Bauru” e veio a realizar-se em 25 de outubro (Centro..., 23 out. 1913; Conferência..., 26 out. 1913). Na ocasião do convite, o francês presenteou seus alunos com a “valiosíssima oferta” de um raro exemplar de seu novo trabalho sobre parasitologia (*Précis de pathologie*, segunda edição), “ainda não saído à luz da publicação” (Souza Campos, 29 set. 1913). É possível conjecturar que esse convite do Caoc representou um movimento de reaproximação da nova gestão desse centro acadêmico com o francês, após as desavenças já comentadas.

Conforme acordado previamente com Carvalho, Brumpt retornou à Europa em novembro, onde permaneceu até março do ano seguinte, quando, no dia 13, desembarcou novamente em São Paulo para dar início a seu segundo ano letivo. Antes do início das aulas, fez uma viagem acompanhado de Bourroul para Salto Grande, na região limítrofe com o Paraná, em razão de “numerosos casos de impaludismo [que] de forma anômala” estavam aparecendo em cafezais da região. Durante a viagem, Brumpt aproveitou a oportunidade “para recolher materiais para seu curso de parasitologia” (Impaludismo, 4 abr. 1914).

Retornou àquele município paulista em maio do mesmo ano, quando fez novas pesquisas parasitológicas e sugeriu medidas profiláticas para o enfrentamento dos três principais problemas de saúde pública locais que “impedem o progresso, que paralisam o desenvolvimento e mesmo em certos casos impedem de colher o que foi semeado nas fazendas”. Eram eles: a ancilostomose (“opilação, amarelão”), para a qual aconselhava “tratar com o thymol todos indivíduos doentes”, “fazer em cada casa uma latrina” e “proibir expressamente que se evacue no chão”; o paludismo (“maleita, sezões”), cujos doentes deveriam ser tratados “energicamente ... por meio de quinina”, e a doença combatida através da “guerra aos mosquitos transmissores”; e, por fim, em menor escala, as úlceras das florestas (“úlcera de Bauru, úlceras da Noroeste, ferida brava”), que, apesar da indefinição de seus vetores, pareciam “ter por causa as numerosas águas paradas ou fontes na floresta”, sendo, portanto, “prudente fugir-se delas” (As fazendas..., 18 maio 1914).

Nesse mesmo mês, Brumpt realizou nova expedição com Alexandrino Pedroso. Dessa vez, foram ao município de Albuquerque Lins para, “na grande floresta, [estudar] os agentes vetores suspeitos da leishmaniose florestal americana” em conhecida zona endêmica (Brumpt, 1934, p.32). A ausência de resultados publicados e o desconhecimento dos vetores desse grupo de doenças, ao menos até a década de 1920, constituem fortes indícios de que essa segunda viagem de estudos das leishmanioses não foi tão proveitosa quanto àquela ocorrida no ano anterior.

No decurso do segundo ano de sua estada em São Paulo, o evento mais significativo realizado pelo parasitologista francês foi a viagem a Lassance (Minas Gerais) “para visitar [Carlos] Chagas e estudar no local a doença que carrega seu nome” (Brumpt, 1934, p.33). Ao que tudo indica, essa visita começou a ser planejada ainda em fins de 1913, quando Arnaldo de Carvalho enviou o seguinte telegrama a Oswaldo Cruz sobre sua ida ao Rio de Janeiro (Figura 1): “Professor Brumpt, chefe da missão organizada pela Faculdade de Medicina de São Paulo para estudo da úlcera de Bauru e moléstia de Chagas, deseja colher em Manguinhos, para onde parte, elementos de trabalho. Peço-vos faciliteis ao distinto cientista o desempenho da tarefa” (Notas, 30 out. 1913). Sendo um dia depois respondido por Oswaldo Cruz: “Terei grande satisfação [em] receber [em] Manguinhos o sr. professor Brumpt, facilitando-lhe todos os elementos [em] nosso alcance” (Notas, 1 nov. 1913).

**Figura 1:** : Émile Brumpt no Pavilhão Mourisco do Instituto Oswaldo Cruz em 30 de outubro de 1913 (DAD/COC-Fiocruz, BR RJCOC 02-10-20-10-017-001)

Em junho de 1914, ao lado de Antonio Carini e João Florencio Gomes (1886-1919), ele visitou o vilarejo onde Chagas realizou sua tríplice descoberta (vetor, parasita e manifestação clínica da tripanossomíase americana). Lá, teve oportunidade de observar numerosos doentes que recebiam tratamento no hospital construído pelo Instituto Oswaldo Cruz (Kropf, 2009), produzir fotografias que serviram para ilustrar as futuras versões de seu tratado de parasitologia (Figura 2) e, acompanhado de Gomes (Figura 3), realizar profícuas prospecções nas regiões adjacentes de Lassance que resultaram na descrição de uma nova espécie de barbeiros, o *Triatoma chagasi*. Esse achado, inclusive, levou-o à suposição de existência de ciclo silvestres da doença de Chagas entre mamíferos selvagens e na caracterização do homem como um reservatório secundário desse tripanossomo, uma vez que o barbeiro foi encontrado em tocas de mocó (*Kerodon rupestris*), em região inabitada, naturalmente infectado pelo *Trypanosoma cruzi* (Brumpt, Gomes, 1914, p.73-77; Brumpt, 1934, p.89).

**Figura 2:** : Crianças doentes da forma crônica da doença de Chagas, fotografadas por Brumpt durante viagem a Lassance (Brumpt, 1936, p.356)

**Figura 3:** : Brumpt no Monte Cabral (800m de altura; 12km de Lassance) onde foi encontrado, em 1913, o *Triatoma chagasi* (Brumpt, 1936, p.349)

Na verdade, o interesse de Brumpt pela tripanossomíase americana (e o estabelecimento de colaborações com pesquisadores brasileiros para seu estudo) precede sua primeira vinda ao país. Em janeiro de 1912, em parceria com o médico baiano Pirajá da Silva, realizou na Faculdade de Medicina de Paris minuciosos estudos e experimentos sobre os ciclos reprodutivo e de vida do *Conorhinus megistus* [atual *Panstrongylus megistus*], espécie vetora da doença de Chagas que havia sido capturada na vila Mata de São João, situada no interior da Bahia. Além disso, demonstraram a existência de abundante quantidade de tripanossomos em suas dejecções e capacidade de infecção em cobaias de laboratório. Essa constatação serviu como forte indício, posteriormente comprovado, de que o procedimento normal de inoculação da doença não se dava pela picada do barbeiro, mas pela contaminação por suas fezes, constituindo a infecção por picada, um procedimento meramente acidental (Brumpt, da Silva, 1912a, 1912b; Brumpt, 1934).

Ao longo das primeiras décadas do século XX, Émile Brumpt se dedicou com grande assiduidade ao estudo da doença de Chagas, contribuindo amplamente para o conhecimento de diferentes facetas dessa tripanossomíase (manifestação clínica, caracterização parasitológica, modo de transmissão, descrição de novas espécies e da distribuição geográfica de seus vetores e hospedeiros intermediários) (Opinel, Gachelin, 2005). A meu ver, no entanto, seu maior e mais duradouro contributo relacionado a essa patologia foi a proposição de um novo método de diagnóstico, concebido durante os últimos meses em São Paulo, que ficaria em voga até o surgimento da biologia molecular: o xenodiagnóstico. De maneira geral, esse método consiste em verificar se barbeiros criados em laboratório (e, portanto, livre de infecções) ficavam infectados após picar indivíduo suspeito de estar com a doença (Brumpt, 1914).

Na verdade, a proposição do xenodiagnóstico para verificação de doenças parasitárias e, em particular, da tripanossomíase americana nada mais foi do que uma releitura, a partir das experiências adquiridas no Brasil, de suas atividades científicas realizadas desde 1904, quando “estudava a evolução dos tripanossomos de peixes e constatou, ... o aparecimento de culturas abundantes de parasitos no tubo digestivo de sanguessugas jovens que haviam se alimentado do sangue de peixes considerados livres de infecção”, quando analisados pela microscopia (Brito, Lima, 2009, p.196).

De acordo com Brumpt, o referido método apresentava numerosas vantagens sobre as outras formas de se chegar ao diagnóstico da doença de Chagas (exame direto do sangue, inoculação em cobaia e produção de culturas), como a maior quantidade de sangue possível de ser absorvida pelo barbeiro quando comparada àquela passível de se examinar “entre a lâmina e a lamínula” de um microscópio, a familiaridade das populações afetadas com o barbeiro e sua “picada indolor” e a inconveniência em se transportar um microscópio a cavalo para longínquas regiões interioranas assoladas pela doença (Brumpt, 1914, p.100, 101; Kropf, 2009).

Assim como os demais trabalhos produzidos durante a estada em São Paulo, o artigo no qual Brumpt propôs o xenodiagnóstico foi originalmente publicado nas páginas dos *Anais Paulistas de Medicina e Cirurgia*. Esse só veio a lume na edição de novembro de 1914; portanto, após sua “brusca partida” em 4 de outubro para auxiliar no esforço de guerra francês (Brumpt, 1914).

O parasitologista prometeu retornar à terra dos bandeirantes tão logo o armistício fosse assinado, deixando, por isso mesmo, seus materiais didáticos e malas com itens pessoais na faculdade, sob a guarda de Celestino Bourroul (Bourroul, 2 maio 1920). Sua promessa, no entanto, não foi cumprida. Em 29 de maio de 1919, Émile Brumpt escreveu para Arnaldo de Carvalho comunicando que não mais reassumiria seu posto em São Paulo. Explicava que, em razão do súbito falecimento de Raphael Blanchard, por insuficiência cardíaca, todos os seus planos “foram modificados”. Após a morte de seu mentor, ele foi unanimemente indicado por seus pares como o sucessor na cátedra de parasitologia da Faculdade de Medicina de Paris.

Com tons amistosos e cordiais, prosseguiu a correspondência dizendo que, “durante toda guerra, por discrição”, não quis importunar Carvalho “falando de questões financeiras”, mas, como a paz estava voltando a reinar, e o custo de vida em Paris aumentando no pós-guerra, solicitava ao diretor que “examinasse com benevolência as indenizações” que considerava justo receber do estado de São Paulo. Ele reclamava o total de oito mil francos, dos quais cinco mil eram referentes à condução de trabalhos práticos em parasitologia durante metade de 1914; mil relativos a adiantamentos feitos em dinheiro na compra de animais e outros gastos durante suas viagens (Bauru e Lassance); e, por fim, dois mil como “indenização parcial pela viagem” de sua família do Brasil para a França. Argumentava que esse valor lhe deveria ser pago pelo fato de o contrato ter sido rompido “por motivo de força maior” e que considerava desnecessário solicitar a intervenção do Ministério das Relações Exteriores para regulação dessa questão. Por fim, concluía a correspondência parabenizando Carvalho pelo “grande sucesso de vossa Faculdade de Medicina” (Brumpt, 26 abr. 1919).

O francês, no entanto, não obteve resposta, o que o levou a enviar nova correspondência em 26 de janeiro de 1920. Em tons menos amistosos e mais diretos, informava que havia acionado o Ministério de Instrução Pública para “alcançar através da hierarquia” os valores reclamados, os quais considerava que “o rico estado de São Paulo poderia facilmente” lhe pagar. Na reclamação enviada ao Ministério, com três páginas, explicava detalhadamente suas demandas e ainda incluía pedido adicional de mais cinco mil francos, caso o material que deixou em São Paulo não lhe fosse devolvido (Brumpt, 26 jan. 1920).

A partir dessa reclamação, o Ministério de Instrução Pública acionou o cônsul francês em São Paulo, que, por sua vez, respondeu afirmando que havia procurado Carvalho e o caso poderia ser considerado encerrado, uma vez que ele lhe mostrara a duplicata do cheque de oito mil francos que havia enviado ao parasitologista francês. Já seus pertences, deixados na faculdade, foram sendo devolvidos aos poucos, na medida em que eram transportados, mala após mala, por pessoas em tráfego entre São Paulo e Paris, a pedido de Bourroul, como ficou claro na correspondência enviada ao francês em maio de 1920:

Meu querido amigo e mestre Brumpt,

Por meu amigo – Senhor Dias – eu vos enviei uma de suas pesadas malas. Como eu não tenho a chave, eu não sei se ele terá algum obstáculo na aduana de Cherbourg ... Você recebeu as outras duas malas, enviadas pelo meu amigo Figueiredo contendo vossas coleções, preparações etc.? (Bourroul, 2 maio 1920).

## Considerações finais

Nas quase três décadas compreendidas entre o imbróglio do desligamento de Émile Brumpt da faculdade paulista e as comemorações do seu jubileu científico em 1948, os contatos estabelecidos entre as partes foram efêmeros, resumindo-se ao acolhimento de alunos de São Paulo para realização de períodos de intercâmbio no seu laboratório parisiense e uma passagem pela cidade, durante escala no regresso de uma viagem que fez a Uruguai e Paraguai, em 1924, quando teve o prazer de rever seus “antigos colegas e alunos da Faculdade de Medicina” (Brumpt, 1934, p.35).

Nesse interregno, o curso médico paulista passou por profundas modificações, deslocando-se da órbita de influência europeia para norte-americana, em grande medida devido aos vultosos aportes financeiros feitos entre 1916 e 1931 pela Fundação Rockefeller, que o transformou em “um dos mais modernos centros de ensino médico da época” (Marinho, Mota, 2012, p.70; Marinho, 2013; Mota, 2005). Em 1934, a faculdade de medicina, junto a outras escolas superiores e institutos de pesquisas já existentes no estado, foi incorporada à recém-criada Universidade de São Paulo (USP), que nas décadas subsequentes construiu e sedimentou sólida reputação acadêmica nacional e internacional (Marinho, Mota, 2012, p.108, 109; Mota, 2005).

Émile Brumpt, por sua vez, consolidou-se como uma das principais lideranças globais da parasitologia. Após tornar-se catedrático de parasitologia na Faculdade de Medicina de Paris, foi nomeado diretor da Escola de Malariologia fundada na Universidade de Paris e, por aclamação, presidente da Société de Pathologie Exotique (1932-1936). Além disso, fundou, com Maurice Langeron e Maurice Neveu-Lemaire, o periódico *Annales de Parasitologie Humaine et Comparée* (1923) e continuou a dedicar-se com grande assiduidade à realização de expedições aos rincões mais distantes do mundo tropical, contribuindo significativamente para a ampliação de sua força profissional.

O início do processo de mitificação de Brumpt como fundador da parasitologia em São Paulo deu-se em 1948, em virtude das comemorações pelos cinquenta anos de trabalhos científicos ininterruptos. Nessa ocasião, a Congregação da Faculdade de Medicina da USP aprovou unanimemente sua condecoração como professor *honoris causa* e a vultosa doação de 50 mil cruzeiros para construção do prêmio internacional Émile Brumpt de Parasitologia, que, anualmente, premiaria o autor do melhor trabalho nesse campo de pesquisa em nível global, servindo assim para a manutenção da memória desse pesquisador que, acometido pela febre maculosa das montanhas, veio a falecer em 1951.

Para concluir, considero necessário destacar que este artigo não tem como objetivo desmerecer a trajetória desse médico francês nem, muito menos, negar seu importante papel na organização da Escola de Medicina de São Paulo, mas, sim, demonstrar que, longe de ser o disseminador da parasitologia em terras tupiniquins, Brumpt foi alguém que soube aproveitar sua estada em São Paulo para contribuir de forma significativa na ampliação dos horizontes desse campo médico-científico que, no início do século XX, ainda se encontrava em construção, contando com investigações muitas vezes feitas em colaboração com membros da comunidade médica local.

## NOTAS

1 É necessário destacar que a institucionalização do ensino superior médico em São Paulo não foi fruto de um processo harmônico, civilizado e hegemônico da classe médica paulista, pois envolveu disputas políticas e projetos divergentes. O grupo responsável pela fundação da Escola de Medicina e Cirurgia de São Paulo como um curso médico com controle estatal saiu vitorioso. Essa não foi, porém, a primeira escola médica a funcionar na cidade. Em 1911, após promulgação da lei Rivadávia Corrêa, que reorganizou o ensino superior no país e permitiu a criação de instituições privadas, foi criada a Universidade de São Paulo, que contava com um curso de medicina e rivalizaria com a faculdade estatal até 1917, quando fechou as portas. Para uma contextualização sobre este assunto, ver Mota (2005), Teixeira (2007) e Maia (2017).

2 Na maior parte da literatura que versa sobre a criação da Escola de Medicina e Cirurgia de São Paulo, é recorrente a informação de que a aula inaugural foi ministrada por Edmundo Xavier (Marinho, Mota, 2012; Mota, 2005; Marinho, 2013, p.113; Fonseca, 2002). Silva (2003, p.177), por outro lado, afirma que “a lição de abertura do curso da Faculdade foi apresentada pelo professor da cátedra de história natural ... Celestino Bourroul”. A mesma informação também é encontrada em reportagem publicada em 2 de junho de 1917, no *Correio Paulistano*, destinada a narrar o histórico desse curso médico (O ensino..., 2 jun. 1917). Dessa forma, acredito que na cerimônia de inauguração houve duas (ou mais) aulas na Escola Politécnica de São Paulo.

3 Sem possuir sede própria, as aulas foram iniciadas na Escola de Comércio Alvares Penteado e na Politécnica. Em 1914, o curso foi transferido para prédio alugado na rua Brigadeiro Tobias, onde permaneceu até 1931 (Mota, 2005; Marinho, Mota, 2012; Fonseca, 2002).

4 Para mais informações sobre Arnaldo Vieira de Carvalho, ver Marinho (2014); Dantes, Silva (2012).

5 Carvalho foi nomeado diretor do curso médico por Francisco Alves de Paula Rodrigues Alves pelo decreto publicado em 7 de janeiro de 1913 (Mota, 2005; Teixeira, 2007; Fonseca, 2002).

6 Consta no artigo 26 da lei de criação da faculdade de medicina a seguinte informação: “Fica o Governo autorizado, para execução desta lei, a despender até a quantia de 500:000$000 (quinhentos contos de réis) com a aquisição do material indispensável à montagem de laboratórios e mais misteres do ensino, abrindo o necessário credito” (São Paulo, 19 dez. 1912).

7 Em sua aula inaugural, Brumpt (21 jun. 1913, p.1) trouxe essa informação ao iniciar sua explanação da seguinte forma: “Sr. Diretor, meus senhores – antes de abordar o assunto do curso que vamos iniciar, permita dizer-vos ainda uma vez que me sinto feliz, como francês, de ser o primeiro estrangeiro chamado a falar nesta escola”. Como veremos, o francês só chegaria em São Paulo em julho de 1913, portanto, três meses após o início do ano letivo. De acordo com Mota (2005, p.190), os primeiros professores estrangeiros que chegaram em São Paulo foram o italiano Alfonso Bovero e o francês Lambert Mayer.

8 Criado em 1902, após três anos de negociações entre a Faculdade de Medicina de Paris e a Union Coloniale, o Instituto de Medicina Colonial tinha por objetivo fornecer estudos sobre doenças exóticas e parasitologia para médicos civis. Esse instituto de pesquisa acabou por tornar-se um grande centro de atração de estudantes latino-americanos interessados no estudo das doenças tropicais (Opinel, 2008, p.391, 392).

9 Em francês os títulos acadêmicos de Brumpt são: *licence ès sciences. sciences naturelles: zoologie, botanique, géologie* (1896); s*ciences physiques: chimie génerale; doctorat ès sciences naturelles* (1901) e *doctorat en medicine* (1906) (Brumpt, 1934, p.5).

10 Nessa e nas demais citações de textos em francês, a tradução é livre.

11 Para melhor contextualização sobre as relações estabelecidas entre a doença do sono e os projetos imperialistas europeus, ver Neill (2012) e Amaral (2012).

12 Para uma robusta análise sobre a trajetória desse médico suíço-brasileiro, ver *Adolpho Lutz: obra completa* (12 volumes), coletânea organizada por Jaime Larry Benchimol e Magali Romero de Sá. Em especial, Benchimol (2005); Benchimol, Sá (2006).

13 Em 20 de julho de 1908, o jornal *O Paiz* reproduziu, na sessão “Comissão de Finanças do Senado”, a autorização de pagamento de 4:200$, em ouro, a Celestino Bourroul como prêmio de viagem (A Comissão..., 20 jun. 1908).

14 De acordo com Marinho (2016, p.116), nessa viagem Celestino Bourroul manteve contato com o Instituto Pasteur de Montpellier e o Instituto de Medicina Colonial, ambos na França, com o Laboratório de Anatomia Patológica da Universidade de Berlim e, sem especificar a instituição, afirmou que foi também a Viena para especialização em clínica médica, radiologia e anatomia patológica.

15 No acervo histórico do Instituto Pasteur de Paris existem duas caixas com documentação relacionada às missões de Brumpt em São Paulo em 1913 e 1914, a saber, BPT.D1 e BPT.D2, nelas podemos encontrar documentações, como livros, artigos científicos, anotações pessoais, cadernos de viagens e, sobretudo, correspondências trocadas com médicos brasileiros. É notória a superioridade de correspondências trocadas com Celestino Bourroul. Além disso, existe também algumas separatas, com o nome do médico paulistano que possivelmente indicam que ele foi o responsável pelo envio de dois livros, escritos em francês, que versavam sobre as características geográficas, culturais e socioeconômicas do estado de São Paulo (Casabona, 1910; L’État..., 1911).

16 No prefácio da segunda edição do *Précis de Pathologie*, em 1913, escrito já em solo brasileiro, Brumpt (21 jun. 1913, p.XV) atribuiu o lançamento em razão de “certos trabalhos de laboratório que eu estive realizando sobre vários Tripanossomos dos mamíferos e em particular sobre o *Trypanosoma Cruzi*”.

17 Como veremos, era grande o interesse de Brumpt pelas leishmanioses encontradas na região sul-americana. Já na primeira edição de seu manual, ele demonstrou grande conhecimento dos trabalhos produzidos por pesquisadores da região, sendo o primeiro autor estrangeiro a validar a *Leishmania braziliensis*, como uma espécie desse gênero de protozoário individualizada. Para mais informações sobre a história das leishmanioses em um contexto não eurocêntrico, ver Benchimol, Jogas Jr. ( 2020) e Benchimol, Peixoto (2022).

18 Essa informação consta em Brumpt (1948, p.49).

19 Para mais informações sobre os projetos que culminaram na criação da Escola de Medicina e Cirurgia de São Paulo, ver Mota (2005), Teixeira (2007) e Silva (2003).

20 Léo Lopes de Oliveira foi escolhido para auxiliar o parasitologista francês como preparador de história natural; no entanto, faleceu em setembro de 1913, logo após a chegada de Brumpt (Homenagem..., 1916, s.p.).

21 Não foram encontradas informações biográficas sobre esse personagem. Silva (2003, p.166, 256) e Teixeira (2007, p.91) fazem referência ao médico Synesio Rangel Pestana como médico da Santa Casa de Misericórdia de São Paulo e membro da Sociedade de Medicina e Cirurgia de São Paulo. É possível que o sobrenome “Rocha” tenha sido grafado de maneira errada na referida reportagem.

22 O conteúdo programático do curso de parasitologia pode ser encontrado em Notícias (1913, p.31-32).

23 Considerado melhor aluno da primeira turma, Ernesto de Souza Campos tornar-se-ia preparador da cadeira de histologia, logo após sua formação e catedrático em 1925. Para mais informações sobre esse personagem, ver Maia (2017).

24 A reunião anterior foi realizada em 15 de julho, data do desembarque do francês no porto de Santos. No início da ata, consta que “o sr. dr. Alves Lima comunicou à casa que, em nome da sociedade, foram dadas as boas-vindas ao notável professor Brumpt, que vem reger a cadeira de história natural da Faculdade de Medicina e Cirurgia de São Paulo” (Ata...., ago.-set. 1913, p.10).

25 Para conhecer melhor a problemática dos chamados sertões paulistas e das doenças dessas paragens, ver Mota (2021). Sobre o genocídio indígena nessa região, ver Dornelles (2017).

26 Para mais informações sobre as pesquisas de Chagas buscando esclarecer o modo de transmissão das leishmanioses, ver Chagas (1972, p.143), publicado originalmente em 1913.
